# The M25 gene products are critical for the cytopathic effect of mouse cytomegalovirus

**DOI:** 10.1038/s41598-017-15783-x

**Published:** 2017-11-14

**Authors:** Ivana Kutle, Sarah Sengstake, Corinna Templin, Mandy Glaß, Tobias Kubsch, Kirsten A. Keyser, Anne Binz, Rudolf Bauerfeind, Beate Sodeik, Luka Čičin-Šain, Martina Dezeljin, Martin Messerle

**Affiliations:** 1 Institute of Virology, Hannover Medical School, 30625 Hannover, Germany; 2 Unit of Mycobacteriology, Institute of Tropical Medicine, 2000 Antwerp, Belgium; 3 Institute for Biomedical and Health Research, University of the West of Scotland, PA1 2BE Paisley, Scotland UK; 4 Centre for Virus Research, University of Glasgow, G61 1QH Glasgow, Scotland UK; 5 Helmholtz Centre for Infection Research, 38124 Braunschweig, Germany; 6 Central Core Unit for Laser Microscopy, Hannover Medical School, 30625 Hannover, Germany

## Abstract

Cell rounding is a hallmark of the cytopathic effect induced by cytomegaloviruses. By screening a panel of deletion mutants of mouse cytomegalovirus (MCMV) a mutant was identified that did not elicit cell rounding and lacked the ability to form typical plaques. Altered cell morphology was assigned to the viral M25 gene. We detected an early 2.8 kb M25 mRNA directing the synthesis of a 105 kDa M25 protein, and confirmed that a late 3.1 kb mRNA encodes a 130 kDa M25 tegument protein. Virions lacking the M25 tegument protein were of smaller size because the tegument layer between capsid and viral envelope was reduced. The ΔM25 mutant did not provoke the rearrangement of the actin cytoskeleton observed after wild-type MCMV infection, and isolated expression of the M25 proteins led to cell size reduction, confirming that they contribute to the morphological changes. Yields of progeny virus and cell-to-cell spread of the ΔM25 mutant *in vitro* were diminished and replication *in vivo* was impaired. The identification of an MCMV gene involved in cell rounding provides the basis for investigating the role of this cytopathic effect in CMV pathogenesis.

## Introduction

Productive infection by many viruses leads to distinct morphological changes in infected cells, collectively known as cytopathic effect (CPE). The most obvious aspect of CPE is cell rounding, while other associated aspects include impact on cell volume, formation of cytoplasmic or nuclear inclusion bodies, cell fusion or aggregation and loss of adherence, and ultimately cell lysis^1^. These alterations of infected cells often result in characteristic plaque formation in a cell monolayer. CPE could be a passive side effect of viral infection, resulting from cellular stress caused by a massive production of virus particles; however, the existence of non-cytopathic viruses indicates that virus replication is not necessarily associated with morphological changes or cytotoxicity. This suggests that generation of CPE by cytolytic viruses rather constitutes an active process and presumably confers benefit to the virus. Morphological changes of infected cells are associated with rearrangement of the cytoskeleton. There is ample evidence that virtually every virus exploits cytoskeleton components to enable entry into cells, to mediate various intracellular transport processes during the infection cycle, and to promote egress of progeny virus^2–5^.

The lytic, productive infection cycle of human cytomegalovirus (HCMV), the prototypic member of the β-herpesviruses, is characterized by typical rounding and enlargement of infected cells^6^. In fact, the latter aspect is so characteristic that the virus received its name based on this property. HCMV is the most prevalent viral cause of malformations in infants and can provoke severe complications in immunocompromised patients (e.g., transplant recipients). With a time period of 48 to 72 hours, the productive infection cycle of HCMV in fibroblasts is considerably longer than that of α-herpesviruses. The rounding of infected cells can occur as early as 6 hours post infection (h p.i.), well before the onset of viral DNA replication and the release of the first viral progeny. At the latest, HCMV-infected cells display a round shape termed early CPE at one day p.i.^7–9^. Induction of cell rounding requires infectious virus and does not occur upon inoculation with UV-inactivated HCMV particles^9^. Furthermore, the effect is sensitive to inhibitors of protein synthesis or transcription, but not to inhibitors of viral DNA replication, suggesting that cell rounding is not induced by a constituent of the virion, but does require *de novo* synthesis of early viral proteins^8^.

Due to the species specificity of HCMV, the importance of morphological alterations of infected cells for viral pathogenesis cannot be tested experimentally *in vivo*. Mouse CMV (MCMV), however, shares many biological characteristics with HCMV, including the property to elicit the typical CPE in infected cells^10,11^. Today, infection of mice with MCMV has become one of the most convenient animal models for studying CMV pathogenesis *in vivo* ^12–14^. The first step towards understanding of the biological relevance of CMV-induced cell rounding is the identification and analysis of MCMV genes mediating this effect.

Here we report on an MCMV mutant that lacks the ability to form typical plaques and to induce cell rounding. The phenotype of the mutant was associated with disruption of ORF M25. Rearrangement of the actin cytoskeleton typically observed in MCMV-infected cells did not take place after infection with the ΔM25 mutant. The 105 and 130 kDa proteins encoded by ORF M25 were able to modify cell morphology when expressed independently of viral infection. While viral gene expression and genome replication of the mutant and of wild-type MCMV proceeded with comparable temporal kinetics, cell-to-cell spread and titers of progeny virus released from cells infected with the mutant were diminished. A first assessment of the ΔM25 mutant *in vivo* revealed that the M25 gene has an important role in viral pathogenesis in the infected host.

## Results

### An MCMV mutant lacking ORF M25 is unable to induce the typical cytopathic effect in infected cells

**Figure 1:**
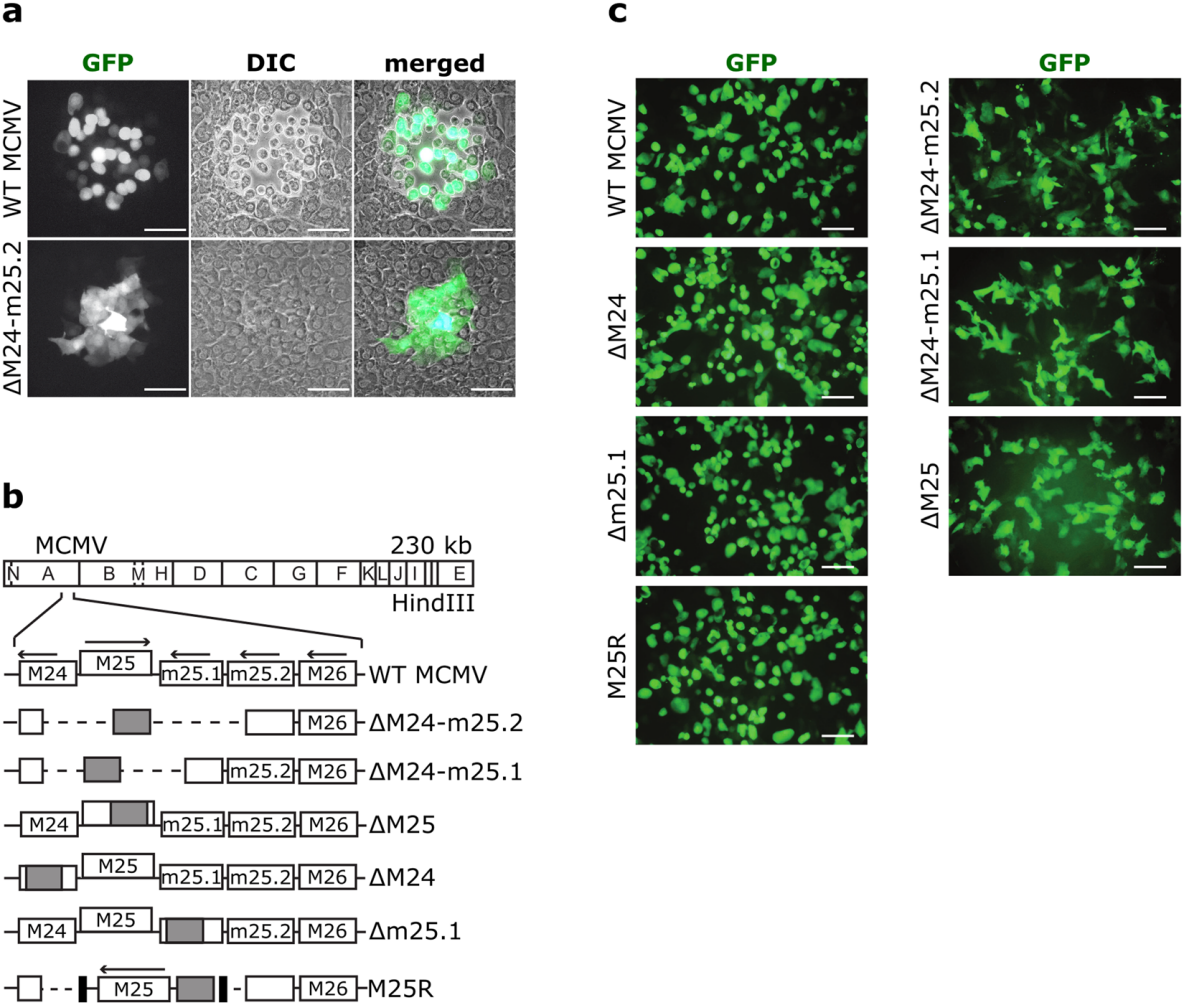
Cytopathic effect induced by WT MCMV or mutant virus. (**a**) Monolayers of C127I epithelial cells infected with WT MCMV or the ΔM24-m25.2 mutant were visualized 72 h p.i. by fluorescence and light microscopy. Foci of infected cells were detected utilizing virus-driven GFP expression. (**b**) The scheme illustrates the genome structure of the mutants. The HindIII cleavage map of the MCMV Smith strain genome is depicted at the top and the genomic region encompassing ORFs M24 to M26 is shown enlarged below. , kanamycin resistance gene; ---, deleted sequences; → , orientation of ORFs; ▪, FRT sites. (**c**) Cells infected with the indicated viruses were examined by fluorescence microscopy 48 h p.i. Scale bars, 100 µm.

Little is known about the involvement of individual MCMV genes in the induction of rounding of infected cells. In order to find viral proteins mediating cytoskeletal changes, a collection of GFP-expressing MCMV deletion mutants^15,16^ was screened, which covered most viral genes dispensable for replication and virion assembly. We identified one mutant, ΔM24-m25.2 that failed to form typical plaques (Fig. 1a). This phenotype was observed on C127I epithelial cells as well as on murine embryonic fibroblasts (MEF) and liver sinusoidal endothelial cells (Supplementary Fig. S1). Whereas most of the cells infected with wild-type (WT) MCMV displayed rounding on day 3 p.i., resulting in disruption of the cell monolayer and plaque formation, the majority of cells infected with the mutant was not rounded at this time point and resembled uninfected cells. Cells infected with the mutant could hardly be discriminated from uninfected cells by light microscopy and were only identifiable by monitoring the GFP expressed from the viral genome (Fig. 1a, lower panel).

The deletion within the genome of the ΔM24-m25.2 mutant affected the four ORFs M24, M25, m25.1 and m25.2 (Fig. 1b). To assign the phenotype to one specific ORF we constructed additional mutants with either a smaller deletion or with disruption of single ORFs. Fibroblasts infected with mutants lacking either ORF M25 alone or additionally adjacent ORFs retained an elongated shape at day 2 p.i. (Fig. 1c, right panels), whereas the viruses with mutations in ORFs M24 or m25.1 induced the typical cell rounding seen with WT MCMV infected cells (Fig. 1c, left panels). This finding strongly suggested that disruption of ORF M25 was responsible for the phenotype of the ∆M24-m25.2 mutant. To verify this result, the M25 ORF (together with appropriate upstream promoter and downstream regulatory sequences) was inserted into the genome of ∆M24-m25.2, resulting in a virus termed M25R (Fig. 1b). This rescue virus induced the same morphological changes in infected cells as WT MCMV (Fig. 1c). Thus, ORF M25 was found to be sufficient to restore the ability for cell rounding.

### ORF M25 encodes two protein species expressed with different temporal kinetics

**Figure 2:**
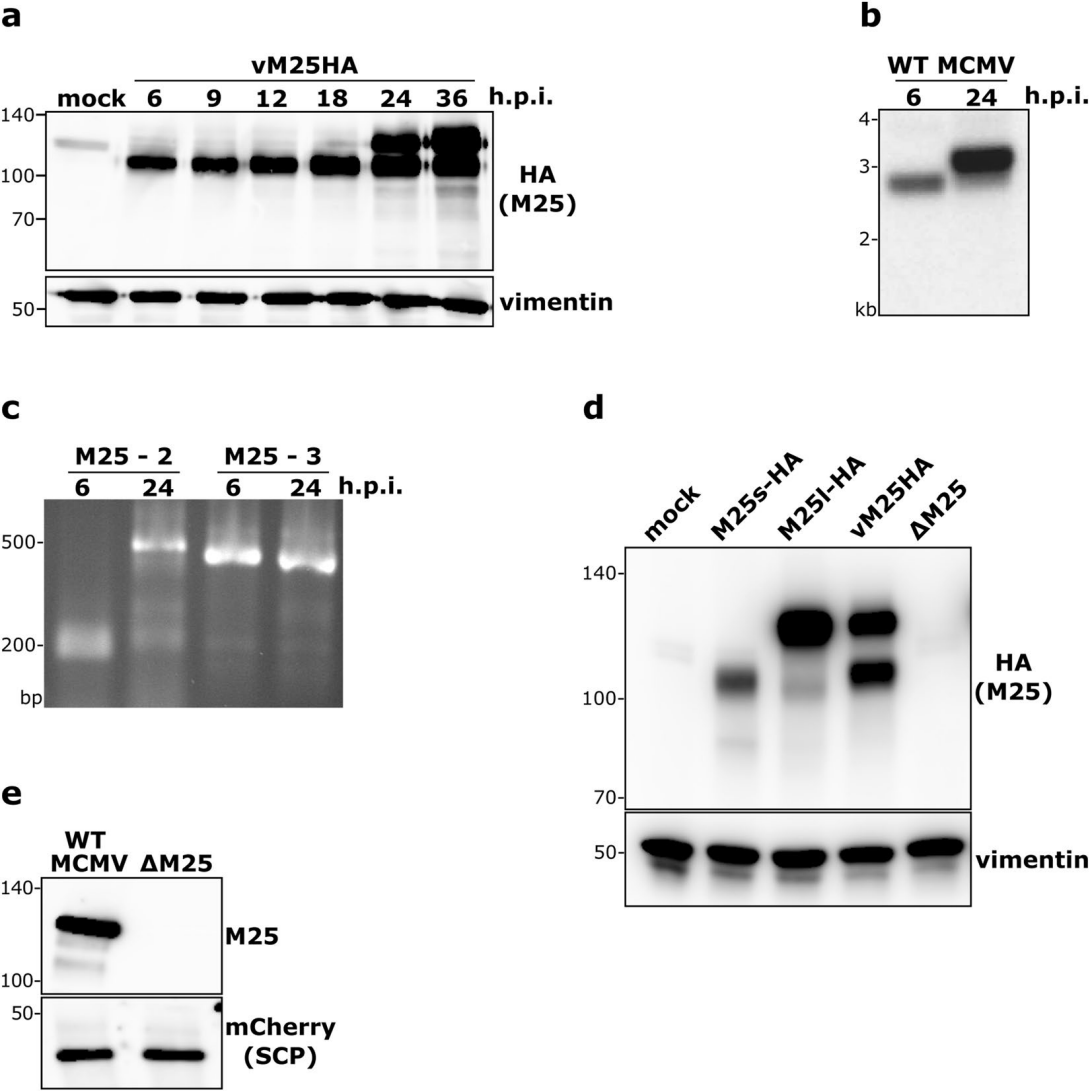
Transcripts and proteins originating from the M25 ORF. (**a**) Lysates of MEF either mock-infected or infected with the vM25HA virus (at MOI 1) and harvested at the indicated time points were subjected to immunoblotting with an HA antibody. Vimentin served as loading control. Protein size markers (in kDa) are indicated to the left. (**b**) Total RNA was isolated from NIH 3T3 cells infected with WT MCMV for 6 or 24 h and subjected to Northern hybridization with a ^32^P-labelled M25-specific probe. (**c**) The 5′- and 3′-ends of the M25 transcripts were determined by RACE using the RNA samples described in (**b**) and primers M25-2 and M25-3. Amplified products were separated by agarose gel electrophoresis and visualized by ethidium bromide staining. Positions of marker bands (**b**,**c**) are indicated to the left. (**d**) Lysates of NIH 3T3 cells prepared 48 h post transfection with plasmids pM25l-HA or pM25s-HA were compared to lysates of NIH3T3 cells infected with vM25HA for 24 h by immunoblotting with an HA-specific antibody. Lysates of mock transfected cells and of cells infected with the ΔM25 mutant for 24 h were included as controls. Vimentin served as loading control. (**e**) Virions were purified from cultures of cells infected with an MCMV variant expressing an mCherry-tagged small capsid protein or the corresponding ΔM25 mutant and subjected to immunoblot analysis with M25- and mCherry-specific antibodies.

It has previously been reported that several antigenically related proteins arise from the M25 gene^17^; however, the relationship of these proteins and their precise origin remained unclear, particularly since only one ~3-kb M25 transcript synthesized with late kinetics has been described^18,19^. To re-examine which proteins are encoded by ORF M25, the sequence for the HA epitope was inserted at the 3′-end of the ORF. In MEF infected with the resulting virus vM25HA a 105 kDa protein was detected at early times of infection (as early as 6 h p.i.) (Fig. 2a). At 24 h p.i., concomitant with the known onset of the late infection phase in this cell type^20^, an additional ~130 kDa band became visible, which further increased in abundance at 36 h p.i. Comparable results were obtained when an M25-specific antibody was used instead of the HA antibody (Supplementary Fig. S2). We tested for different posttranslational modifications of the M25 proteins, and did not observe a change in the mobility of the M25 proteins (Supplementary Fig. S2). Thus, the size difference between the 105 and 130 kDa M25 proteins is not due to the posttranslational modifications examined.

By Northern Blot analysis and 5′-RACE experiments we discovered a novel 2.8-kb transcript expressed with early kinetics (Fig. 2b,c), which has not been described before. By sequencing of the 5′-RACE product the 5′-end of this transcript was mapped to nucleotide position 26,504 of the MCMV strain Smith genome (compare Supplementary Fig. S3). Moreover, we confirmed the expression of an abundant 3.1 kb mRNA late in infection – in agreement with data of Dallas *et al*.^18^ – and determined its 5′-end at nucleotide position 26,191. Mapping of the 3′-ends (to position 28,956) indicated that the two transcripts are co-linear at this end, sharing the poly(A) signal sequence previously described for the late transcript^18^.

We reasoned that the first ATGs present on the respective mRNAs (corresponding to the 2^nd^ and 6^th^ ATG of the M25 ORF as annotated by Rawlinson *et al*.^21^) serve as initiation codons for the synthesis of the 130 and 105 kDa M25 proteins, respectively. To test this idea, two expression plasmids (pM25l-HA and pM25s-HA) were constructed that comprised the ORFs starting at those ATGs. The immunoblot in Fig. 2d revealed that the 130 and 105 kDa proteins detected in cells transfected with the plasmids pM25l-HA and pM25s-HA, respectively, migrated with identical mobility as the corresponding M25 proteins present in vM25HA-infected cells. Further support for the hypothesis came from the analysis of the viral mutant vM25GFPstop in which we inserted the GFP ORF immediately downstream of the sixth ATG of the M25 ORF (Supplementary Fig. S2). The 28 and 48 kDa protein species, detected by immunoblotting with a GFP-specific antibody in cells infected with this mutant (Supplementary Fig. S2), are in line with the expected use of the proposed start codons.

### The subcellular localization of the M25 proteins changes during the course of infection

**Figure 3:**
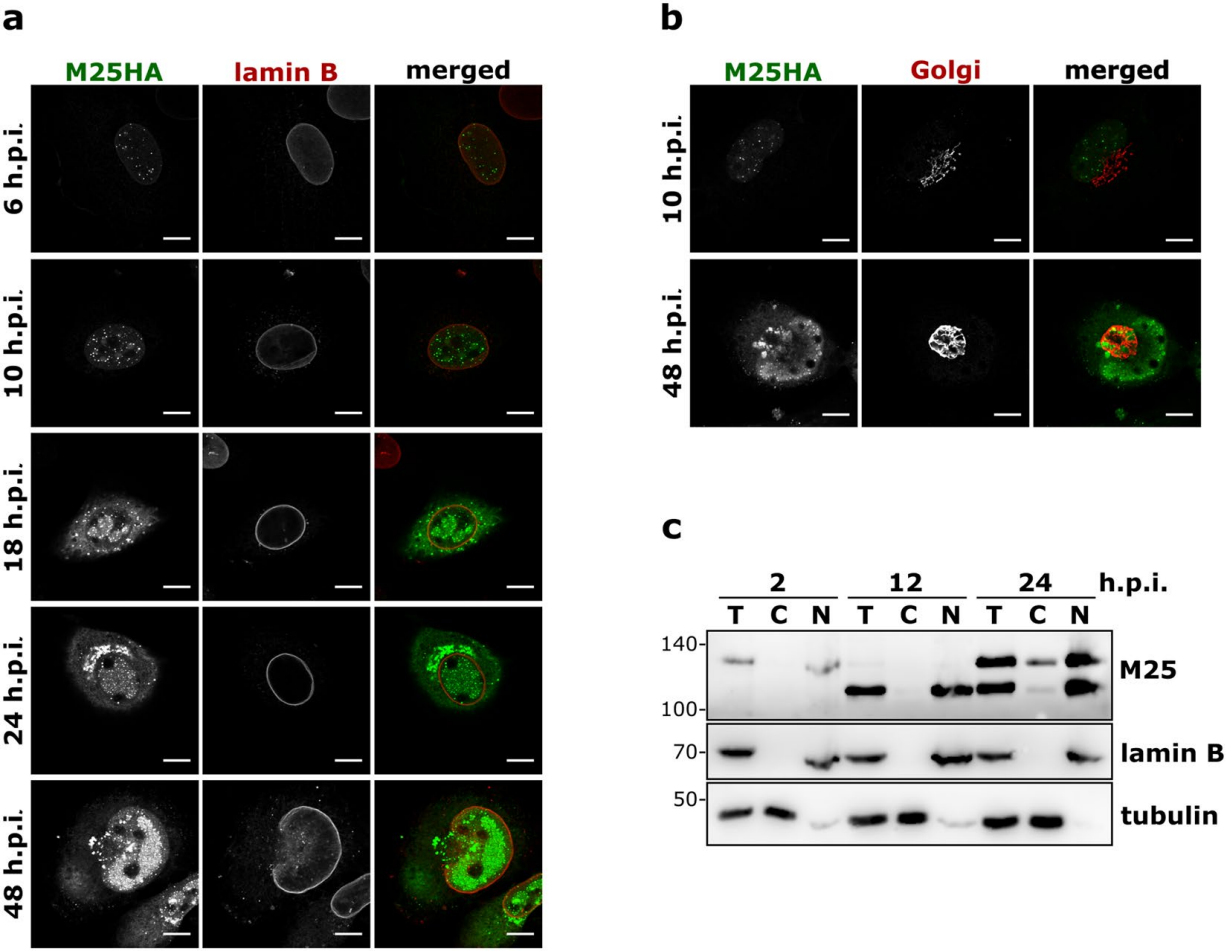
Subcellular localization of M25 proteins during the MCMV infection cycle. (**a,b**) MEF infected with the vM25HA virus for the indicated time periods were analyzed by confocal microscopy after labeling with antibodies specific for the HA epitope, lamin B and the Golgi protein GM130. Size bar, 10 µm. (**c**) NIH 3T3 cells were infected with the vM25HA virus. At the indicated time points either total cell lysates (T) or cytoplasmic (C) and nuclear (N) fractions were prepared and analyzed by immunoblotting with the M25-specific antibody. Fractionation was verified for lamin B and β-tubulin.

When we examined infected cells by immunofluorescence microscopy, dot-like structures were detected in the nucleus of infected cells at early time points (6 and 10 h p.i.) (Fig. 3a). From 18 h p.i. on, the M25 proteins became also visible in the cytoplasm enriched in dot-like structures, and in the nucleus the M25 proteins were present within distinct patches. Late in infection, at 24 and 48 h p.i., additionally accumulation of the M25 proteins was observed within the cytoplasm, juxtaposed next to the nucleus. When co-staining the infected cells with an antibody against the Golgi matrix protein GM130, we found that the cytoplasmic accumulations of the M25 proteins were surrounded by structures that contained this Golgi marker protein (Fig. 3b). This is characteristic for the cytoplasmic virion assembly compartment formed in CMV-infected cells^22,23^.

To learn which of the M25 proteins were present in the cytoplasm or nucleus, lysates of vM25HA-infected cells were separated into cytoplasmic and nuclear fractions and analyzed by immunoblotting. At 2 h p.i. small amounts of the 130 kDa M25 protein were found in the nuclear fraction (Fig. 3c). The 130 kDa M25 protein detected at this very early time point probably originates from incoming virus particles entering the cells, as this M25 protein species has been described as a tegument component of MCMV virions^17,24^. When examined at 12 h p.i., the 105 kDa M25 protein was present exclusively in the nuclear fraction. Late during the infection cycle (24 h p.i.) both the 105 and the 130 kDa protein were found in the nucleus, and especially the 130 kDa protein was also present in the cytoplasmic fraction (Fig. 3c). Accumulation of the 130 kDa M25 protein within the cytoplasm is consistent with the expected incorporation into virions^17^.

### Lack of M25 proteins leads to smaller virus particles

To check for the presence of M25 proteins in virus particles, virions were purified by ultracentrifugation from the supernatant of cell cultures infected with WT MCMV or the ΔM25 deletion mutant and examined by immunoblotting with an M25-specific antibody. The immunoblot in Fig. 2e confirmed that predominately the 130 kDa M25 protein species is incorporated into MCMV virions.

**Figure 4:**
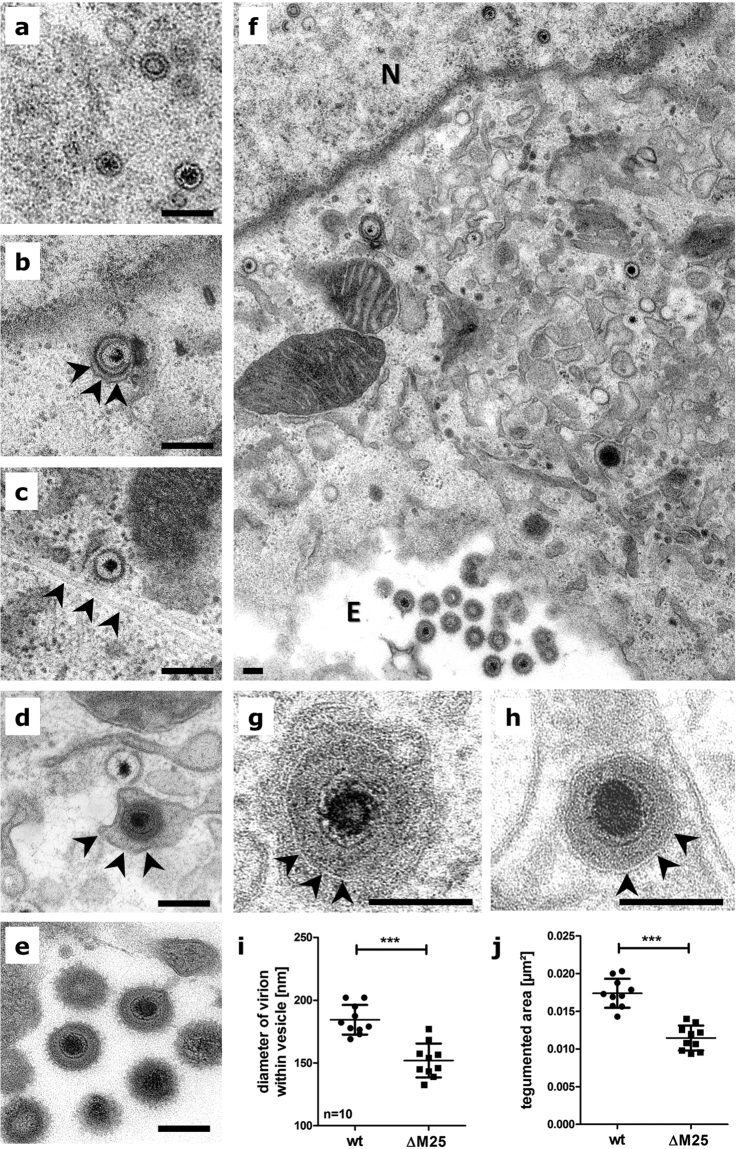
Virions of the ΔM25 mutant are smaller. Transmission electron microscopy of ultrathin-sections of MEF infected with the ΔM25 mutant (**a**–**f**, and **h**) or WT MCMV (**g**). Cells infected with the mutant virus (**f:** overview, N = nucleus, E = extracellular space; (**a–e**,**h**): higher magnifications) assemble nuclear capsids (**a**), primary enveloped capsids (**b**, arrowheads indicate the outer nuclear membrane), cytosolic capsids in proximity to microtubules (**c**, arrowheads point at a microtubule), secondary enveloped virus particle (**d**, arrowheads indicate the vesicle membrane) and extracellular virions (**e**). Diameters (**i**) and the sectioned tegumented area (**j**) of cytoplasmic virions were measured for 10 virions each (**g**, WT MCMV; **h**, ΔM25 mutant; arrowheads indicate the viral membrane). Means ± SD are depicted and statistical analysis was done using the paired t-test. ***P < 0.001. Scale bars, 200 nm.

Next, we analyzed the formation of virus particles in the absence of M25 proteins. Ultra-thin-sections of MEF infected for 24 h with the ΔM25 mutant or WT MCMV were prepared and subjected to transmission electron microscopy. In cells infected with the ΔM25 mutant (Fig. 4a–f) or the WT virus (data not shown) all stages of viral morphogenesis were observed, capsid assembly in the nucleus represented by A, B, and C capsids (Fig. 4a), primary envelopment at the nuclear membrane (Fig. 4b), cytosolic capsids sometimes associated with microtubules (Fig. 4c), secondary envelopment in the cytoplasm (Fig. 4d), and extracellular progeny virions attached to the plasma membrane (Fig. 4e). However, when the diameters of enveloped mature particles present within cytoplasmic vesicles were measured and compared (Fig. 4g,h and i), it became obvious that the virions of the mutant were significantly smaller and this was due to a thinner tegument layer (Fig. 4j). For HCMV it has been reported that lack of one tegument protein can impair the recruitment of other tegument proteins into the virions^25,26^. The amounts of the MCMV tegument proteins M82 and M83 were however not markedly altered in virions of the ΔM25 mutant (Supplementary Fig. 2). This suggests that the smaller size of ΔM25 virions is primarily due to the lack of the 130 kDa M25 tegument protein, consistent with the high abundance of this M25 protein in virions of WT MCMV^17,24^.

### Viral gene expression and DNA replication of the ΔM25 mutant and WT MCMV are comparable

**Figure 5:**
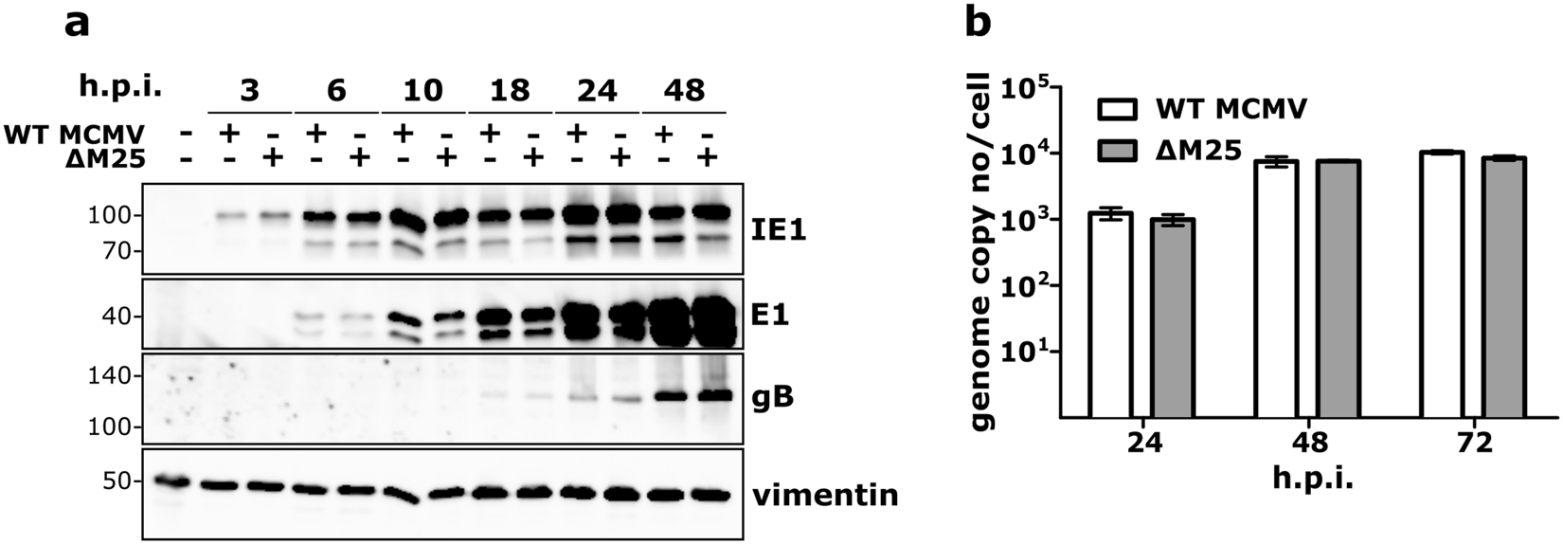
Viral gene expression and genome replication are not affected by the lack of M25 proteins. (**a**) NIH 3T3 cells were either mock-infected or infected with the indicated viruses (MOI 3.) At the indicated time points lysates were prepared and analyzed by immunoblotting using antibodies specific for the viral immediate-early protein IE1, early protein E1, or glycoprotein B. Vimentin served as loading control. (**b**) MCMV genome copy numbers present in NIH 3T3 cells (infected as described in (**a**)) were measured by qPCR at the indicated time points. Graph shows means ± SD of 2 to 3 independent experiments.

To gain insight into how the lack of the M25 proteins affects the viral life cycle, the expression of the IE1, E1 and gB proteins that are representative for the different kinetic classes (immediate-early, early and late) was examined. In cells infected with ∆M25 or WT MCMV, little differences in the amounts of these viral proteins were seen at the individual time points p.i. (Fig. 5a). This finding indicates that the absence of the M25 proteins has no influence on the expression of other viral proteins. Analysis of viral DNA replication revealed the presence of substantial genome copy numbers as early as 24 h p.i., with a further increase by 48 and 72 h p.i. (Fig. 5b). Yet again, the amounts of viral DNA were similar for the ∆M25 mutant and WT MCMV, implying that the M25 proteins are not involved in viral DNA replication.

### The ∆M25 mutant displays diminished cell-to-cell spread and grows to lower titers

**Figure 6:**
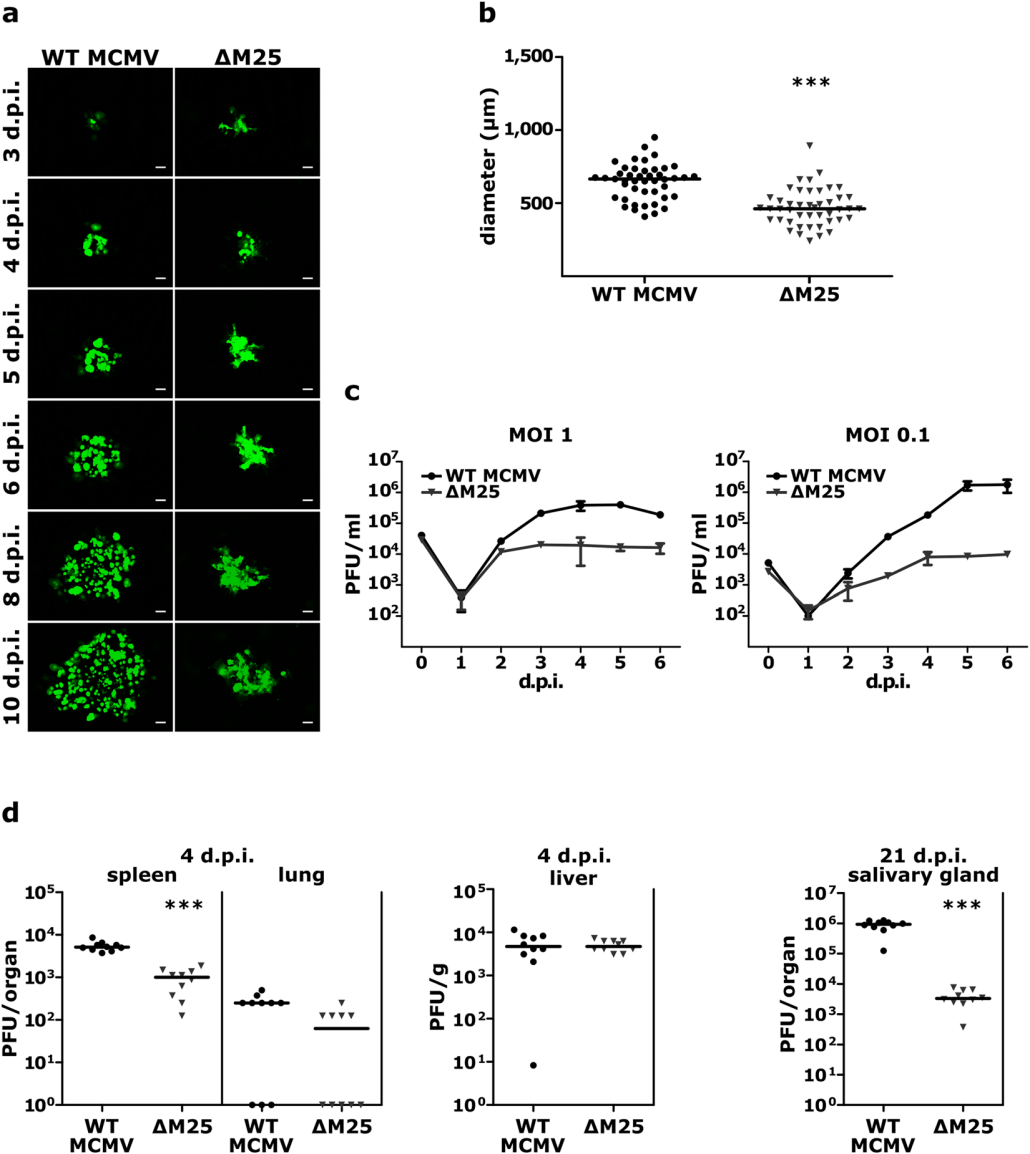
Diminished cell-to-cell spread and virus yields of the ∆M25 mutant. (**a**) Foci formation of infected cells was monitored for 10 days by fluorescence microscopy of MEF monolayers infected with WT MCMV or the ∆M25 mutant at low MOI. Scale bars, 100 µm. (**b**) Areas of infected cells were measured for WT MCMV (n = 45) and the ∆M25 mutant (n = 45) on day 5 p.i. Infected cells were identified based on virus-driven GFP expression. Bars in the graphs represent medians and statistical analysis was performed by Mann-Whitney two-tailed test. ***P < 0.001. (**c**) MEF were infected with the indicated viruses at an MOI of 0.1 or 1. Culture supernatants were collected at indicated days after infection and titers quantified by plaque assay. Data points represent means ± SD of triplicate samples. (**d**) BALB/c mice were infected intraperitoneally with MCMV-GFP-ie1/3 (WT) or MCMV-GFP-ie1/3_ΔM25 (ΔM25), and virus titers in liver, spleen and lungs at day 4 p.i. and in salivary glands at day 21 p.i. were determined by plaque assay. Each dot represents the value for one animal (n = 10 for each group) and bars indicate medians. Statistical significance was calculated by Mann-Whitney two-tailed test. ***P < 0.001.

Following infection of cell monolayers at low multiplicity, we observed that the areas of GFP-positive cells were smaller for the ΔM25 mutant than for the parental WT MCMV (Fig. 6a), suggesting that cell-to-cell transmission of the ΔM25 mutant was less efficient. Measurement of the areas of infected cells showed that the foci formed by the ΔM25 mutant on day 5 p.i. were significantly smaller than the plaques produced by WT MCMV (Fig. 6b). To examine whether the release of infectious progeny into cell culture supernatant was also reduced, single-step and multiple-step growth curve analyses were performed with MEF and liver sinusoidal endothelial cells, and the growth kinetics of the ∆M25 virus and WT MCMV were compared (Fig. 6c, Supplementary Fig. S4). Following infection at an MOI of 1, the yields of the mutant in MEF on day 5 were ~20-fold reduced in comparison to the parental MCMV, and upon infection at MOI 0.1 the difference was ~200-fold (Fig. 6c). Additional analysis revealed that also the yields of intracellular virus were approximately one order of magnitude lower for the ∆M25 mutant when compared to WT MCMV (Supplementary Fig. S4). Taken together, formation of mature, infectious particles was reduced in the absence of the M25 proteins, lower titers of viruses released from ΔM25 infected cells were observed and cell-to-cell spread of the ΔM25 mutant was diminished.

To analyze the importance of the M25 gene for replication of MCMV *in vivo*, we used an MCMV variant (MCMV-GFP-ie1/3) displaying wild-type properties and the corresponding ΔM25 mutant (MCMV-GFP-ie1/3_ΔM25). We constructed and used these new viruses because the variants used in the previous experiments carry a mutation in the mck2 gene^27^ and are known to be attenuated *in vivo*. BALB/c mice were infected intraperitoneally with 2 × 10^5^ PFU of the indicated viruses, and viral titers in spleen, lungs and liver were determined on day 4 and in salivary glands on day 21 p.i. (Fig. 6d). The viral titers for the ΔM25 mutant in spleen on day 4 p.i. were approximately one order of magnitude lower than for the parental virus and there was a trend towards lower titers in lungs (the amount of virus progeny in lungs was still low at this time point and in some mice below the detection limit). In liver, similar titers were measured for the two viruses on day 4 p.i. Three weeks after infection the titer of the ΔM25 mutant in the salivary glands was more than two orders of magnitude lower than the titer of the parental virus (Fig. 6d), an observation which is typical of attenuated viruses. We concluded that the ΔM25 mutant can establish infection in different organs, and that attenuation becomes particularly manifest in the considerably reduced titers in salivary glands.

### Cytoskeletal changes in ΔM25 and WT MCMV-infected cells

**Figure 7:**
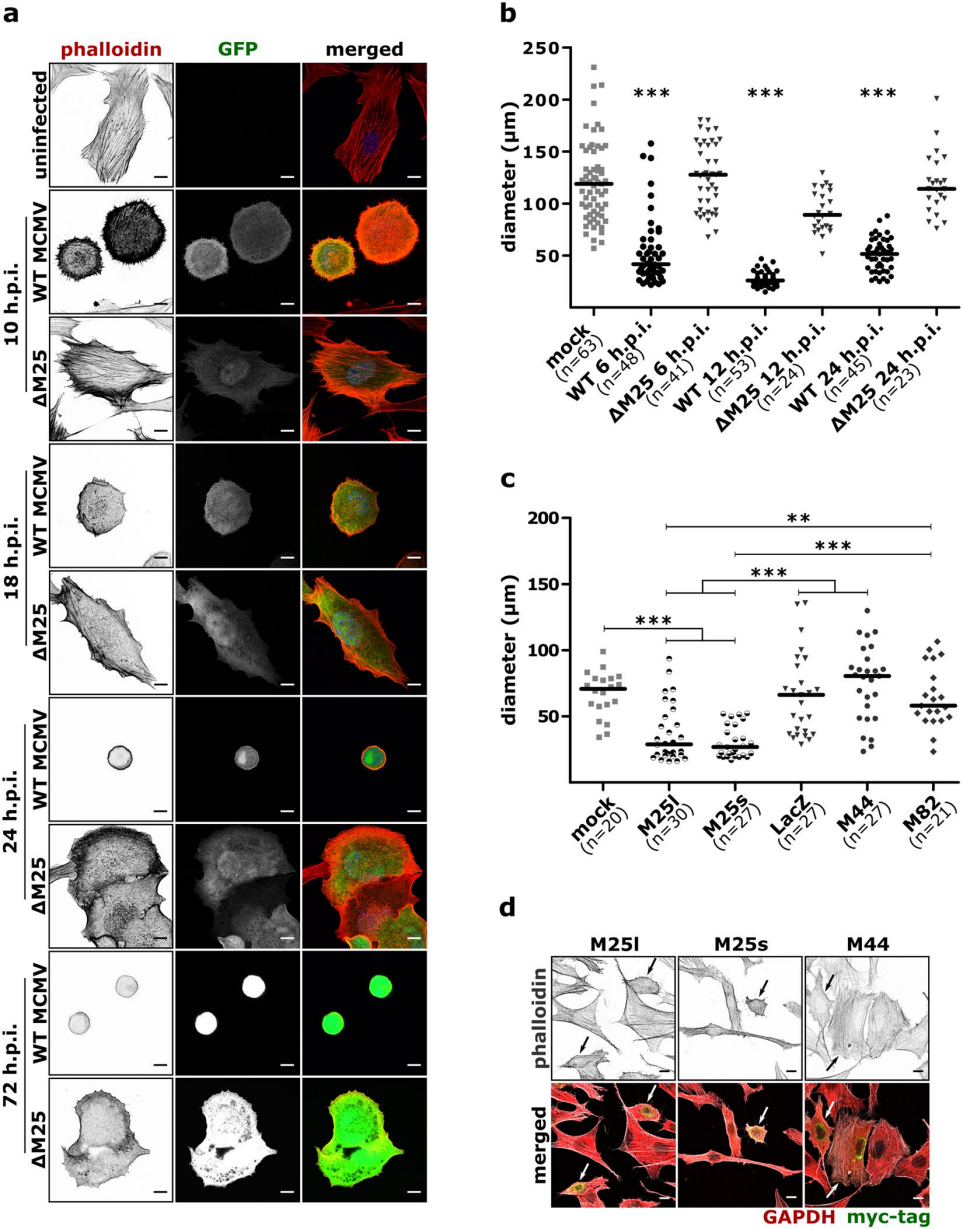
Morphological changes in WT MCMV and ∆M25 mutant infected cells and in cells transfected with M25 expression plasmids. (**a**) NIH 3T3 cells were infected with WT MCMV or the ∆M25 mutant or were mock infected. Cells were fixed at the indicated time points, labelled with TRITC-phalloidin and analyzed by confocal microscopy. Positive staining of actin appears black or dark grey. Infected cells are distinguished by virus-driven GFP expression. Size bars, 10 µm. (**b**) Diameters of the infected cells (treated as indicated in (**a**)) were determined. (**c**,**d**) NIH3T3 cells were either mock transfected or transfected with plasmids pM25l, pM25s and control plasmids encoding the viral proteins M44 or M82 or *E.coli* ß-galactosidase (lacZ). After 24 h cells were fixed, labeled with Alexa Fluor 488 phalloidin, and antibodies directed against GAPDH and the myc epitope, and examined by confocal microscopy. Transfected cells were identified by the myc signal. Size bars, 10 µm. (**c**) Cell diameters as determined 24 h after transfection. Bars represent medians. Statistical significance was tested using the Kruskal-Wallis test followed by Dunn’s post hoc test. ***P < 0.001; **P < 0.01. (**d**) Representative images of cell cultures transfected with M25l, M25s or M44 plasmids. Phalloidin (actin) staining is depicted in the top row and merged signals of phalloidin, GAPDH and myc labeling are shown below. Transfected cells (myc + signal in green) are indicated by arrows.

One of the characteristics of the ΔM25 mutant is the apparent absence of the rounding of infected cells. Therefore, we examined the impact of WT MCMV on cell morphology in more detail and compared it to that of ΔM25 mutant. Cells were fixed at different time points after infection, actin filaments were labeled with TRITC-phalloidin and visualized by fluorescence microscopy. Non-infected fibroblasts showed an elongated and spread morphology and an extensive network of actin fibers was visible in most of the examined cells (Fig. 7a). Conversely, as early as 6 h p.i. WT MCMV-infected cells started to round and to detach from neighboring cells (Supplementary Fig. S5). These morphological changes coincided with disassembly of actin fibers spanning the cell, and enrichment of cortical actin with clearly visible short retraction fibers in most of the infected cells (10, 18 h p.i.) (Fig. 7a). As infection proceeded, cells became nearly spherical with actin fibers rearranged exclusively at the rim of the cells. On the contrary, ΔM25-infected cells retained the flattened morphology throughout the course of infection, with cell-cell contacts preserved for most of the cells. Compared to WT MCMV-infected cells, loss of transverse actin fibers in ΔM25-infected cells was delayed (Fig. 7a, time points 10 and 18 h p.i.). At late stages of infection (48, 72 h p.i.) the majority of ΔM25-infected cells displayed protrusions resembling lamellipodia and membrane ruffles.

Next, we quantified the cell rounding at various stages of infection by measuring the longest diameter (maximum feret) of individual cells. At all time-points examined (6, 12, 24 h p.i.) sizes of ΔM25-infected and non-infected cells were comparable (Fig. 7b), while WT MCMV-infected cells displayed smaller diameters.

To investigate whether cell rounding can be induced solely by the M25 proteins, we measured sizes of cells transfected with the expression plasmids pM25l and pM25s encoding the 130 and 105 kDa M25 proteins, respectively. Expression of either M25 isoforms diminished cell size compared to mock transfection or transfection with control plasmids encoding LacZ or the viral proteins M44 or M82 (Fig. 7c). Closer inspection of the cells revealed that the morphological changes of M25 transfected cells (Fig. 7d) were not as strong as upon WT MCMV infection. We concluded that the M25 gene products are able to alter cell shape, yet additional viral factors may contribute to the formation of the characteristic CPE of MCMV-infected cells.

## Discussion

In this study we identified and characterized an MCMV mutant that lacks the ability to elicit cell rounding. We linked this phenotype to ORF M25 and confirmed that the two M25 proteins expressed differ in their temporal expression kinetics and change their subcellular localization over the course of the infection cycle. A newly detected 2.8 kb M25 mRNA defines the coding sequence for the 105 kDa M25 protein and explains its early expression kinetics, whereas a 3.1 kb late mRNA gives rise to the 130 kDa M25 protein. Transfection of cells with plasmids encoding the M25 protein species led to reduction of cell size, connecting them with the morphological changes. Other salient features of the ΔM25 mutant were smaller size of virus particles and growth to lower titers. A first assessment of the replication capacity of the ΔM25 mutant *in vivo* suggests that the M25-mediated functions are relevant for viral pathogenesis.

After assigning the phenotype of the mutant to ORF M25 we examined the proteins encoded by this ORF, and detected two protein species of sizes similar to the ones previously described by Wu *et al*.^17^ for the MCMV strain K181. Expression of the 105 kDa M25 protein was noticed slightly earlier than before^17^ – probably due to the high detection sensitivity of the HA antibody used. The newly discovered 2.8 kb M25 mRNA now explains the appearance of the 105 kDa M25 protein, and we confirmed that the 3.1 kb mRNA encodes the 130 kDa M25 protein. Wu *et al*.^17^ reported on an additional, antigenically related 90 kDa M25 protein, yet using pulse-chase experiments they did not detect a precursor-product relationship to the other M25 products. In some of our experiments we observed a 90 kDa M25 protein species, too, especially when cells were harvested by trypsin treatment. This implies that the 90 kDa protein is a proteolytic degradation product, whose appearance mainly depends on the conditions applied for sample preparation.

The most striking feature of the ΔM25 mutant is the failure to induce rounding of infected cells. We and our colleagues analyzed dozens of MCMV mutants^15,16,28^, including several ones with reduced replication capacity, and never observed a comparable loss of cytoskeletal remodeling for other mutants. This strongly suggests that M25 proteins have a major impact on formation of the CPE. The temporal kinetics observed for cell rounding implies that early in infection the 105 kDa M25 protein is responsible for inducing the effect, maintained by the 130 kDa protein in the late phase. The mechanism used by the M25 proteins to mediate cytoskeletal remodeling will be the subject of further research. Since we did not observe colocalization of M25 proteins with components of the cytoskeleton, the underlying mechanism is probably indirect and may involve targeting of signaling pathways that induce actin changes. Moreover, it is likely that in infection the M25 proteins act together with other viral proteins to accomplish the CPE, because the morphological changes seen in cells transfected with M25 expression plasmids did not fully mimic the CPE observed in infected cells. As a first step towards unraveling the mode of action of the M25 proteins, we are currently in the process of identifying M25 interaction partners.

Other characteristics of the ΔM25 mutant were growth to lower titers and reduced cell-to-cell spread. Cytoskeletal changes elicited by a series of viruses^5,29^ have been associated with increased release of virus progeny and cell-to-cell spread^30–32^. Moreover, several herpesvirus tegument proteins have been implicated in virion trafficking, virion egress and transmission to neighboring cells^33–35^ – besides their role in secondary envelopment and virion assembly. It is entirely possible that the M25 proteins have a similar function, although it remains to be determined whether the phenotypes are interconnected or whether the M25 proteins exert these tasks independently. CMVs are highly cell-associated, and within the infected host direct cell-to-cell transmission appears to be the sole mechanism for viral dissemination^36,37^. Cytoskeletal remodeling is therefore of particular relevance for CMVs to accomplish cell-to-cell spread. At first glance cell rounding seems to be counterintuitive in this respect, however, this could be misleading and cytoskeletal rearrangements may indeed be essential to establish new and specific cell-cell contacts, for instance by forming nanotubes or filopodia as described for HSV-1^38^. Despite the importance, little is known about the mechanisms driving cell-to-cell transfer of CMVs^36^. Hence, the ΔM25 mutant and the M25 proteins provide useful tools for analyzing this subject.

Although the electron microscopy experiments did not reveal an obvious deficiency in secondary envelopment of capsids and virion formation, less infectious particles of the ΔM25 mutant were found inside of cells as well as extracellularly. Since there was no difference in viral gene expression and genome replication between ΔM25 and WT MCMV infected cells, we concluded that the maturation of ΔM25 particles is impaired. A reasonable explanation is the absence of the M25 tegument protein, particularly as it has been identified as an abundant constituent of MCMV virions^24^. Accordingly, it is not surprising that particles of the ΔM25 mutant are of smaller size. In HCMV, it has been found that tegument proteins build an interaction network^39,40^. Absence of one tegument protein could readily affect incorporation of other tegument proteins, and this has been observed for pUL25 in HCMV virions lacking pp65^25,26^. On the other hand, the tegument of different CMVs displays remarkable plasticity, resulting – for instance - in little alteration of the protein composition of pp65-deficient rhesus CMV virions^41^ or in maintenance of HCMV particle size when pp65 is missing^25,26^. Interestingly, the absence of the M25 tegument protein in virions of the ΔM25 mutant did not affect the abundance of the M82 and M83 tegument proteins, which are homologs of major HCMV tegument proteins (pp65 and pp71). A more comprehensive analysis is needed to examine how the absence of other virion-associated proteins is influenced when M25 is missing.

A first assessment of the properties of the ΔM25 mutant *in vivo* revealed growth to lower titers in most organs except liver. In view of the phenotype of the ΔM25 mutant *in vitro*, the attenuation *in vivo* was not unexpected. Surprising was, however, the different outcome in liver which could be due to infection of specific cell types in this organ. Following systemic infection, in liver most progeny virus is produced by hepatocytes^42^, whereas in spleen the major targets of productive infection are endothelial cells^42,43^. It will be of interest to investigate whether there is a cell type-specific difference in the requirement of M25 for the MCMV infection cycle. Notably, a cell-type specific function in cell-to-cell spread has been reported for the UL51 tegument protein of HSV-1^34^ and this protein has recently been implicated in modulation of cell shape as well^35^.

Colonization of salivary glands occurs during a second viremic phase after initial replication of MCMV in primary infected organs. In view of the lower titers in most of these organs, it is not surprising that the attenuation of the ΔM25 mutant manifests in even higher titer differences in salivary glands. Moreover, cytoskeleton remodeling may change the migratory capacity of infected myeloid cells that are known to spread MCMV within the body of infected mice^37,44,45^, thereby contributing to the observed phenotype. In summary, MCMV M25 mutants will be highly useful tools for investigating the connection between virally induced cytoskeletal changes and pathogenesis in a natural virus host model.

## Materials and Methods

### Cells

Mouse embryonic fibroblasts (MEF) prepared as described^12^ and C127I cells (ATCC CRL-1616) were propagated in DMEM supplemented with 10% FCS (PAN Biotech). NIH 3T3 fibroblasts (ATCC CRL-1658) were cultured in DMEM with 5% NCS (Sigma-Aldrich). Conditionally immortalized MEF^46^ (kindly provided by T. May) were maintained in DMEM with 10% FCS, 1% NEAA, 0.1% β-mercaptoethanol, and 2 μg/ml doxycycline (Sigma-Aldrich), and conditionally immortalized liver sinusoidal endothelial cells (LSEC) were propagated as described^47^. All culturing media were supplemented with penicillin/streptomycin. Cell cultures were regularly checked for the absence of mycoplasma contamination using a PCR-based mycoplasma detection kit (Minerva Biolabs). For transfection NIH 3T3 cells were seeded in 6-well plates (2 × 10^5^ cells/ well) on coverslips coated with fibronectin (Sigma-Aldrich). 20 h later 6 µg of plasmid DNA complexed with 6 µl of Lipofectamine Plus and 6 µl of Lipofectamine LTX (both from Invitrogen) were added and cells further incubated in medium with 2% NCS, followed by exchange with DMEM/5% NCS after another 6 h.

### Viruses and infection studies in cell culture

The BAC-derived MCMV-GFP strain^48^ served as WT MCMV in this study. All MCMV variants were propagated on MEF, purified and stored as described^12^. Titers were determined by plaque assay on MEF and growth curve analysis was performed as described in Supplementary Methods. For all kinetic experiments, cells were inoculated with virus resuspended in CO_2_-independent medium (Life Technologies) containing 0.1% (w/v) BSA at the indicated MOI for 2 h at 4 °C to allow virus binding. Then, the inoculum was replaced by complete medium and cells were further incubated at 37 °C in a 5% CO_2_ humidified atmosphere. For screening of mutants, cells were infected at MOI 3 followed by centrifugal enhancement (800 × g for 20 min). Plaque morphology was analyzed in cultures of C127I cells, MEF or LSEC infected with highly diluted viruses. To measure plaque areas, images of plaques were acquired with an AxioObserver Z1 microscope (Zeiss) equipped with a digital camera (Axiocam HRm) and controlled by Axiovision software (version 4.8.2). Plaque sizes (Feret diameters) were measured using Image J (v1.51j; NIH) by manual selection of GFP-positive areas using the polygonal selection tool.

Virions of the MCMV mutants S-mCherry-SCP and S-mCherry-SCP-ΔM25 were enriched by centrifugation of cell culture supernatants as described^12^. Then, the pellet was resuspended in VBS buffer (50 mM Tris-HCl pH 7.8, 120 mM KCl, 6 mM EDTA) overnight at 4 °C. Following water bath sonication at 4 °C (10-s pulses with amplitude of 60% until the sum of the applied energy was 4 kJ), the suspension was overlaid on a continuous 10 to 40% (w/v) Nycodenz (Axis Shield) gradient in VBS buffer and centrifuged at 20,000 rpm for 105 min at 4 °C (Optima L-90K Ultracentrifuge, SW32 rotor). The virus bands were detected by light scattering, collected and frozen at −80 °C.

### Primers and plasmid construction

The sequences of all primers used in this study are listed in Supplementary Table S1. The M25 ORF as defined by Rawlinson *et al*.^21^ plus sequences for a C-terminal HA tag were PCR-amplified with primers M25f and M25HAr and cloned into the expression vector pIRES2AcGFP1 (Clontech). The ORF present in the resulting plasmid pM25-HA was shortened by PCR-based mutagenesis^49^ using primers M25ATG2 and HMIEPr or M25ATG6 and HMIEPr such that the ATGs at nucleotide (nt) positions 26,270 and 26,540 of the MCMV genome^21^ served as start codons in plasmids pM25l-HA and pM25s-HA. The same ORFs were also cloned into plasmid pcDNA4-myc-6xHis (ThermoFisher), leading to pM25l and pM25s. pcDNA4-myc-6xHis-based vectors carrying the ORFs for *E.coli* galactosidase (lacZ) or the MCMV proteins M82 and M83 served as controls. The integrity of all cloned sequences was verified by sequencing.

### Construction of recombinant MCMV genomes

Mutagenesis of MCMV BACs was performed by red-α, -β, -γ-mediated recombination in *E.coli* as previously described^50^ or by *en passant* mutagenesis^51^ and further detailed in Supplementary Methods. BAC DNA was isolated from *E. coli* cultures using an alkaline lysis protocol^50^. The integrity of the mutated BACs was verified by restriction enzyme analysis and by sequencing of the modified regions. MCMV recombinants were reconstituted by electroporation of NIH 3T3 cells with BAC DNA as described^50^ or by transfection using JetPEI transfection reagent (Polyplus-transfection).

### RNA analysis

Total RNA was isolated from MCMV infected cells using the RNeasy kit (Qiagen). For Northern blot analysis, 10 µg of RNA per lane were separated on a denaturing formaldehyde-1% agarose gel, transferred onto a nylon membrane by capillary transfer and immobilized by UV cross-linking. Pre-hybridization was done with hybridization buffer (6 × SSC, 0.1% Ficoll, 0.1% polyvinylpyrrolidone, 0.1% BSA, 50% (v/v) formamide, 1% SDS, 50 μg/ml salmon sperm DNA) for 2 h at 42 °C. An M25-specific probe was generated by PCR using MCMV BAC DNA as template and primers M25pf and M25pr (Supplementary Table S1) and was radiolabeled with α- ^32^P-dCTP using the DecaLabel DNA labeling kit (Fermentas). Hybridization was carried out overnight at 42 °C. Membranes were washed for 15 minutes each in 2 × SSC, 2 × SSC-0.1% SDS, 1 × SSC-0.1% SDS and 0.1 × SSC-0.1% SDS, and finally exposed to Kodak X-Omat-AR films.

Mapping of the 5′- and 3′-ends of the M25 transcripts was performed with the 5′-/3′-RACE kit (Roche) using M25 gene-specific primers (Supplementary Table S1). The amplified DNA products were separated by agarose gel electrophoresis, eluted and sequenced.

### Immunoblotting

Transfected or infected cells were lysed in RIPA buffer (150 mM NaCl, 50 mM Tris-HCl pH 7.4, 1% NP-40, 0.1% SDS, 0.5% Na-deoxycholate) supplemented with Protease Inhibitor Cocktail Set III (Calbiochem). Purified viral particles were lysed in boiling Laemmli sample buffer. Subcellular fractionation was performed following a published protocol^52^ (Supplementary Methods). Proteins were separated by SDS polyacrylamide gel electrophoresis and transferred onto nitrocellulose membranes (Amersham). Membranes were blocked with 5% non-fat milk in TBS with 0.1% Tween for 1 h at room temperature, and incubated with the following antibodies overnight at 4 °C: rabbit anti-HA (Sigma-Aldrich), rabbit anti-HA (Cell signaling), anti-IE1 MCMV (CROMA101; Capri, Rijeka, Croatia), anti-M112-113/E1 MCMV (CROMA103; Capri), anti-M25 MCMV (M25C.01; Capri), anti-gB MCMV (M55.01; Capri), anti-M82 (Capri), anti-M83 (Capri), anti-lamin B (Santa Cruz), anti-tubulin B (Calbiochem), anti-vimentin (Cell signaling), anti-GAPDH (Cell signaling), anti-GFP (Cell signaling), anti-mCherry (Abcam), anti-Phospho-p44/42 MAPK (Erk1/2) (Thr202/Tyr204) (Cell signaling), anti-GlcNac (Cell signaling). Following incubation with HRP-coupled secondary antibodies (Dako) at room temperature for 1 h, signals were visualized by chemiluminescence (SuperSignal West Femto maximum-sensitivity substrate; ThermoFisher) using an LAS-3000 imaging system (Fujifilm).

### Immunostaining and fluorescence microscopy

Cells were grown on glass coverslips coated with fibronectin (Sigma-Aldrich). At appropriate times after infection or transfection cells were fixed with 3% paraformaldehyde for 20 min, permeabilized with 0.3% Triton X-100 for 5 min and blocked with 0.2% gelatine in PBS or 5% goat serum in PBS for 30 min. Labeling was done with primary rat anti-HA (Roche), rabbit anti-HA (Cell Signaling), rabbit anti-GM130 (Abcam), goat anti-lamin B (Santa Cruz), rabbit anti-GAPDH (Cell Signaling) and mouse anti-myc (Santa Cruz), and secondary Alexa Fluor 647-conjugated anti-rat, Alexa Fluor 647-conjugated anti-rabbit, Alexa Fluor 488-conjugated anti-rabbit, Alexa Fluor 647-conjugated anti-mouse, Alexa Fluor 568-conjugated anti-rabbit, Alexa Fluor 546-conjugated anti-goat (each from Molecular Probes). The actin cytoskeleton was labeled with TRITC-phalloidin (Santa Cruz) or Alexa Fluor 488 phalloidin (Invitrogen). For analyzing M25 localization or changes of the actin cytoskeleton, images were acquired using the confocal laser scanning microscopes LSM 510 Meta (Zeiss) or TCS SP8 (Leica Microsystems) with 63×/1.40 oil immersion objectives. Images were further processed in Adobe Photoshop 6.0 or Image J (v 1.51j). Documentation for feret diameter measurement of infected or transfected cells was acquired using the AxioObserver Z1 microscope (Zeiss) and the equipment described above. Infected cells were detected by virus-driven GFP expression and transfected cells by anti-Myc labeling. Measurement was performed using the particle analysis feature of the Image J software.

### Transmission electron microscopy

MEF infected with WT MCMV or the ΔM25 mutant were fixed 24 h p.i. with 2% glutaraldehyde in cacodylate buffer (130 mM (CH_3_)_2_AsO_2_H, pH 7.4, 2 mM CaCl_2_, 10 mM MgCl_2_) for 1 h at room temperature. Cells were contrasted with 1% (w/v) OsO_4_ in cacodylate buffer (165 mM (CH_3_)_2_AsO_2_H, pH 7.4, 1.5% (w/v) K_3_[Fe(CH)_6_]) followed by 0.5% (w/v) uranyl acetate in 50% (v/v) ethanol overnight. The cells were embedded in plastic (29.19 g Epon 812, 12.66 g DDSA, 16.58 g MNA, 0.75 ml DMP30; Serva, Heidelberg, Germany) and 50 nm ultrathin sections were cut parallel to the substrate. Images were acquired with Tecnai G2 at 200 kV or Morgani at 80 kV transmission electron microscopes (FEI, Eindhoven, The Netherlands). For the measurement of virion size and tegument layer, images of fully assembled cytoplasmic virions were analyzed. Virion diameter and the sectioned tegument area were measured using an Image J plugin.

### Quantification of viral genomes

DNA was extracted from infected cells with High pure viral nucleic acid kit (Roche) and genome quantification was done by qPCR as described^53^ using fluorescent probe technique (Supplementary Methods).

### Infection of mice

All animal experiments were performed in compliance with the German animal protection law (TierSchG) and were approved by the Niedersächsische Landesamt für Verbraucherschutz und Lebensmittelsicherheit under permit number 33.19-42502-04-14/1712. Mice were housed and handled in accordance with the recommendations and guidelines of the Federation of European Laboratory Animal Science Associations (FELASA) and the national animal welfare body (GV-SOLAS). 8 week-old BALB/c mice were infected by intraperitoneal injection of 2 × 10^5^ PFU of MCMV_GFP-ie1/3 or MCMV_GFP-ie1/3-ΔM25. 4 or 21 days p.i. mice (10 per group) were sacrificed, and spleen, lungs and salivary glands were harvested as entire organs, while liver was cut into pieces (90–150 mg). All organs were stored at −80 °C until analysis. Viral titers of organ homogenates were determined by plaque assay.

### Statistical analysis

Statistical tests were performed using GraphPad Prism software (GraphPad Software, La Jolla, CA). The non-parametric Mann-Whitney test (two-tailed) was used to compare data of two groups. Comparison of more than two groups was done by one-way ANOVA using the non-parametric Kruskal-Wallis test and Dunn’s post hoc test to analyze differences between specific groups. Virion size and tegument layer area were statistically analyzed by paired t-test. Differences were considered statistically significant for *P* values < 0.05.

### Data availability

The datasets generated during the current study are available from the corresponding author.

## Electronic supplementary material

Supplementary Information
