# Supplementary material for: The M25 gene products are critical for the cytopathic effect of mouse cytomegalovirus
Source: Sci Rep. 2017 Nov 14;7:15588. doi: 10.1038/s41598-017-15783-x (PMC5686157; doi:10.1038/s41598-017-15783-x)
Supplement: Supplementary file 1 — Supplementary Information [file 41598_2017_15783_MOESM1_ESM.pdf]

The M25 gene products are critical for the cytopathic effect of mouse cytomegalovirus

Ivana Kutle, Sarah Sengstake, Corinna Templin, Mandy Glaß, Tobias Kubsch,

Kirsten A. Keyser, Anne Binz, Rudolf Bauerfeind, Beate Sodeik, Luka Cicin-Sain,

Martina Dezeljin & Martin Messerle

Supplementary Information

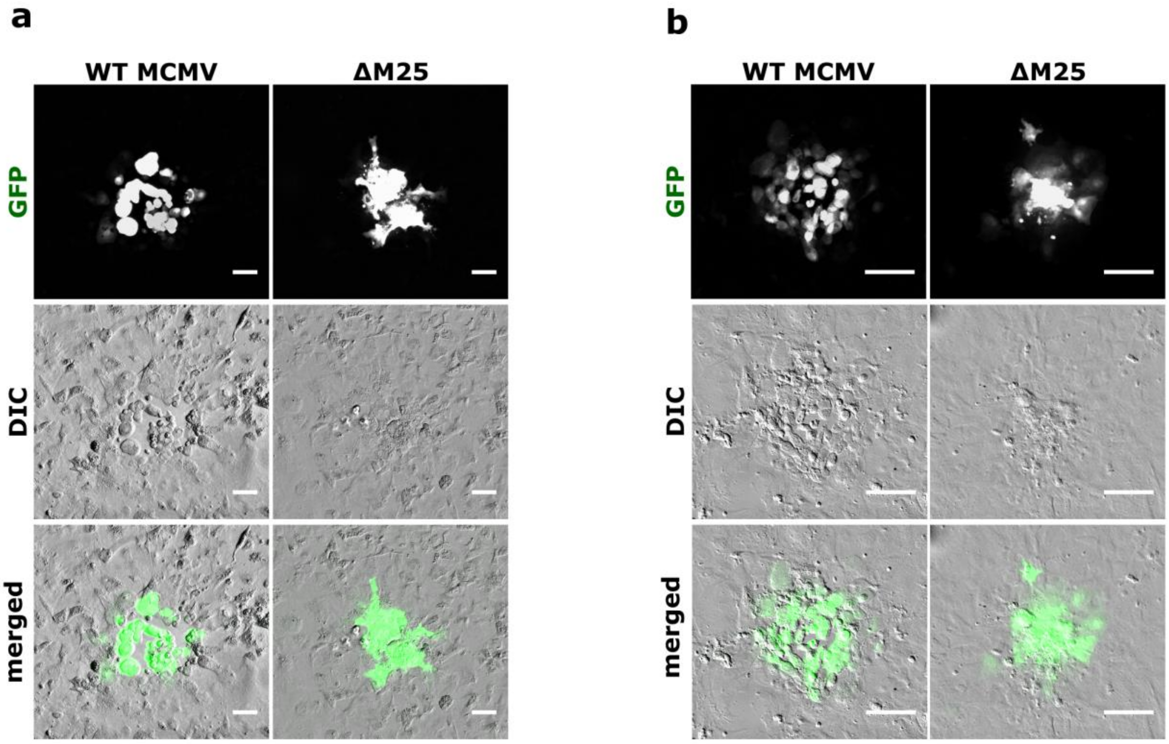

Figure S1. Foci formation of WT MCMV or ΔM25 infected cells. Monolayers of MEF (**a**) or liver sinusoidal endothelial cells (**b**) were infected with the indicated viruses at low MOI. Images of infected cells were taken 5 days p.i. utilizing viral GFP expression. Scale bar, 100 μm.

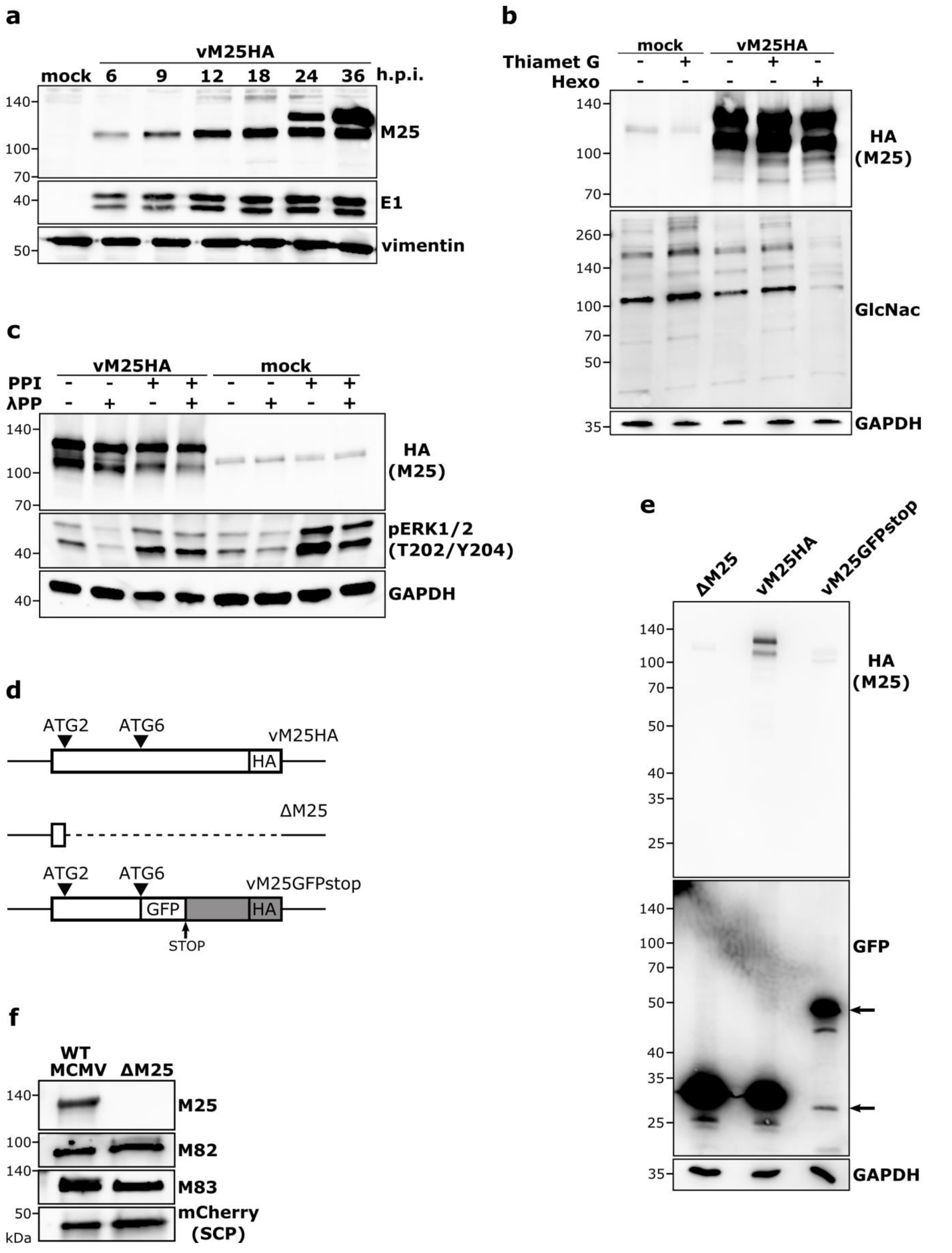

Figure S2. Characteristics of the M25 proteins. (**a**) MEF either mock infected or infected with the vM25HA virus at MOI 1 for the indicated time periods were harvested and lysates were subjected to immunoblotting with an M25-specific antibody. Vimentin served as loading control and the viral E1 protein as marker of

viral infection. (**b, c**) Different mobility of the two major M25 proteins is not due to O-glycosylation and phosphorylation. (**b**) Cells were either treated with O-GlcNAcase inhibitor Thiamet G to enhance global levels of O-glycosylation or after lysis at 24 h p.i. lysates were treated with β-N-Acetylhexosaminidase$_f$ (Hexo) to remove O-GlcNAc. The M25 isoforms were detected using the HA antibody, and O-glycosylation using a GlcNAc-specific antibody. GAPDH was used as loading control. (**c**) Lysates were prepared from cells 24 h p.i. with or without phosphatase inhibitors (PPI) and indicated samples were additionally treated with λ phosphatase (λPP). Immunoblotting was performed with the indicated antibodies. pERK served as control for the treatments and GAPDH as loading control (**d**) The scheme illustrates the structure and the modification of the M25 ORF within the genome of the indicated viruses. The second and sixth ATG are the predicted start codons for synthesis of the 105 and 130 kDa M25 proteins. Insertion of the GFP ORF is expected to give rise to two GFP products and to disrupt expression of HA-tagged M25 protein species. (**e**) Protein species encoded by the modified M25ORF of the vM25GFPstop virus. MEF were infected with the ΔM25, vM25HA or vM25GFPstop viruses at MOI 1 and lysed 36 h p.i. Immunoblotting was performed using HA and GFP antibodies. GAPDH served as loading control. The molecular mass of the protein species indicated by the arrows is in line with initiation of translation at the predicted start codons (ATG2 and ATG6). Please note that the strong GFP signal for the ΔM25 and vM25HA viruses results from a GFP expression cassette, which is driven by the strong HCMV major immediately early promoter and inserted into the m128 (ie2) locus[1]. The vM25GFPstop virus is devoid of this GFP expression cassette. (**f**) Presence of M82 and M83 tegument proteins in virions of the ΔM25 mutant. Virions of the indicated viruses were purified as described in Fig. 2f and presence of the indicated proteins was analyzed by immunoblotting.

```
26001 CCCCGGCCGG CGGCATGAGC CAGTTCGTAC AGCACGTCGC TGACCGTGGC CTCGGCGTCG TCCACCAGCG GCTCGCACGC GGCGTCGAAC CGCGAGCCGA

26101 TTTTCCCCGT CGAATCCATC TCCGCATCCG AACCCTGCCC AGAGATGGAG ACGTGCAACT ATATAAAATC ACGAGAGCCG GGGTCTGCGT GCCAGCCGTC

                                                                        M  N R R  S S K  D R R M
26201 CTATTCCCCG TGCCGGCGCG TCCCTTTCGA CCTCGAACGC CTGTCCGAGA ACCCCCACTC ACCCGAACGA TGAACCGTCG ATCCTCCAAG GACCGTCGTA

   F V T  D D S  S D D D  D D D  V M I  M D P P  E T T  S S S  S S A L  A T A
26301 TGTTCGTCAC CGACGACTCG TCGGATGATG ACGACGACGA TGTGATGATC ATGGACCCCC CGGAGACGAC GTCGTCGTCG TCATCGGCGC TGGCGACGGC

A  G I R  G V P H  A P A  S N S  A T A A  A A S  E S T  Y K P L  S I P  S E
26401 GGCGGGGATA CGGGGCGTTC CCCATGCCCC CGCCTCCAAC TCCGCCACGG CGGCGGCGGC CTCCGAGAGC ACATACAAGC CATTGAGCAT CCCGTCTGAG

E  L N G E  E E E  R D E  E D  M  S R D  G P R  R H S Q  D D D  F T Y  A D P A
26501 GAACTAAACG GCGAAGAGGA GGAGAGAGAC GAAGAAGACA TGTCACGCGA CGGACCCCGC CGGCACAGCC AGGACGACGA CTTTACTTAT GCGGACCCGG

D  V R L  R A  M M G N  R Y G  G Q S  R S A A  T A G  A A S  R N D S  G S V
26601 CCGATGTGAG ACTGCGGGCG ATGATGGGCA ATAGGTACGG CGGACAGAGT CGGAGTGCGG CGACAGCGGG AGCAGCGTCC CGGAATGATA GTGGAAGCGT

S  P V T  L F D E  D G Y  A I I  P D P P  T S R  D D S  R H V V  V D D  D D
26701 GTCGCCCGTG ACCCTTTTTG ATGAGGACGG ATACGCGATA ATCCCCGACC CACCTACTTC GCGCGACGAT TCGCGACACG TCGTCGTGGA CGACGACGAC

D  G D Y D  S H Y  G V  M T V A  P S P  P K L [P R K S  R P S] T K K  S A E E
26801 GACGGAGATT ATGATTCACA CTACGGCGTG ATGACAGTCG CTCCGAGCCC ACCCAAACTG CCACGCAAGA GTCGACCTTC GACCAAAAAA TCGGCGGAGG

K  Q S S  T A G  R S R G  R S T  A R R  T P K K  A Q E  T A P  A A A A  G S
26901 AAAAGCAATC TTCCACCGCC GGCCGGTCCA GAGGCCGCTC GACCGCACGC CGGACCCCGA AGAAGGCTCA AGAGACCGCT CCGGCGGCCG CGGCGGGCTC

G  A R Q  K Q R Q  Q Q Q  Q Q P  P R R Q  S Y H  P P P  D Y P P  P P P  P V
27001 CGGAGCTCGC CAAAAACAAC GCCAGCAGCA GCAACAACAG CCTCCACGCC GCCAATCTTA CCACCCTCCA CCCGATTATC CTCCCCCACC TCCGCCCGTA

Q  A T V S  R P L  P R T  P N A N  D D D  D D D  D N D E  P G P  S N T  R R G
27101 CAGGCGACTG TGTCGCGTCC TCTCCCCAGG ACCCCGAACG CCAACGACGA CGATGACGAC GATGACAACG ACGAGCCGGG TCCGAGCAAC ACACGCCGCG

K  T P C  R R V  D H T E  N N H  L Y E  T P I S  A T A  M V  I D I E  D D E
27201 GCAAAACGCC CTGCCGTCGT GTCGATCACA CGGAGAATAA TCATCTATAC GAGACCCCGA TATCCGCCAC CGCCATGGTG ATCGATATCG AAGATGACGA

D  E E T  G G A A  D D A  S I V  V E D D  D E E  E E N  D C E E  I C D  G E
27301 GGACGAAGAG ACCGGCGGCG CCGCCGACGA CGCGTCTATC GTCGTCGAAG ATGATGATGA AGAAGAGGAG AACGATTGCG AGGAGATCTG CGACGGGGAA

E  E P A A  A A A  A A A  A S S S  T P H  R T Q  P L P V  P P S  S P R  I T R
27401 GAAGAGCCGG CAGCAGCAGC AGCAGCAGCG GCGGCATCGT CGTCGACTCC TCATCGCACC CAGCCTCTGC CCGTCCCACC GTCGTCTCCG CGCATCACGC

E  L G F  L P G  V V S G  Q D A  R F I  A A C L  H H S  H A P  Q V D I  I N
27501 GCGAGCTCGG GTTCCTGCCC GGTGTAGTGA GCGGTCAGGA CGCCAGATTC ATCGCGGCGT GCCTCCACCA CTCGCACGCG CCGCAGGTCG ACATCATAAA

T  C Y P  M P P  Y T L  D A L  S E P V  L T K  K A L  R C A G  V L R  P V I
27601 CACCTGCTAT CCGATGCCGC CGTACACCCT AGACGCGCTA TCTGAGCCGG TCCTGACCAA GAAGGCGCTG CGCTGCGCGG GGGTGCTGCG GCCCGTCATC

K  L A I L  V N Y  Y C V  G I G R  L A R  A R A  L S K D  L M  T P P  R I E T
27701 AAGCTAGCCA TCCTGGTGAA TTACTACTGC GTAGGGATCG GGCGTCTGGC CCGTGCTCGC GCGCTGTCCA AAGATCTGAT GACCCCGCCG CGCATCGAGA

L  R R R  L E G  L L P Q  Q T S  P S P  P M C  L R V  L G R  L N I T  A A Q
27801 CGCTGCGCCG CCGTCTGGAG GGCCTCCTGC CCCAGCAGAC GTCGCCCAGC CCTCCGATGT GCCTGCGGGT GCTCGGCCGC CTGAACATCA CCGCGGCGCA

H  K A S  C D T I  D Q L  M K  P M Q E  R E R  R R Q  K T Q C  A Q L  F R S
27901 GCACAAGGCC AGCTGCGATA CGATCGACCA ACTGATGAAA CCGATGCAGG AACGCGAGCG CCGCCGACAG AAGACGCAGT GCGCACAGTT GTTCCGCAGC

K  N L L F  S P P  R F T  R E G A  K T L  Y  M  R N I K  I L N  S D E  E D T T
28001 AAGAACCTGC TGTTCAGTCC CCCGCGTTTC ACCCGCGAGG GGGCGAAGAC CCTGTACATG CGGAACATCA AGATCCTGAA CAGCGACGAG GAGGATACGA

L  N L V  M T  L N P H  P T R  E D V  L N D A  I F C  L S L  G N F V  Y N F
28101 CGCTGAACCT AGTGATGACC CTGAATCCGC ATCCCACACG GGAAGATGTG CTGAACGACG CGATCTTTTG CCTGTCGCTG GGAAACTTTG TGTACAACTT

S  R A L  E E L R  G  M  I R C  Q F E D  L T E  T L Y  A A Y Y  Q C P  I M R
28201 CTCGCGCGCG CTCGAAGAAC TGCGCGGAAT GATCAGATGC CAGTTCGAGG ACCTGACCGA GACCCTGTAC GCGGCCTACT ATCAGTGTCC CATAATGAGA

D  D Y R V  L C S  E V A  N E I T  S P R  E D G  Q G L S  A L C  R R S  L A F
28301 GACGACTACC GCGTGCTGTG CTCCGAGGTG GCGAACGAGA TCACGTCGCC CCGTGAAGAC GGACAGGGCC TGTCCGCGTT GTGCCGCCGC AGCCTCGCTT

A  R R C  Y N E  G V F F  S P S  Y V K  Y L I K  C A A  M E  E A G F  E G Y
28401 TCGCGCGCCG CTGCTACAAC GAAGGCGTGT TTTTCTCACC GTCGTACGTC AAGTATCTGA TCAAGTGCGC GGCCATGGAA GAGGCAGGCT TCGAAGGCTA

S  L E S  A A R S  L A N  P D I  F R P L  P D E  S S A  R R  M  L R R  T I H
28501 CTCGCTCGAG TCGGCCGCGA GATCTCTGGC GAACCCAGAC ATCTTCCGCC CGCTGCCTGA CGAGAGTAGC GCCCGCCGCA TGCTGCGCCG CACGATACAC

F  V R V D  G T P  S S S  R Q I P  T T H  I P T  H A N Y  E L F  L Q A  S R M
28601 TTCGTGCGCG TCGACGGGAC ACCCTCGTCT TCCAGACAGA TCCCCACGAC CCACATCCCA ACACACGCCA ACTACGAGCT GTTCCTGCAA GCGTCTCGCA

I  V P Q  Q Q Q  S R R S  S T P  P P S  S S P P  P P A  A G G  P K Y S  K R
28701 TGATCGTTCC GCAGCAACAG CAGTCACGCA GAAGCAGCAC CCCGCCACCA TCATCCTCTC CTCCTCCCCC GGCCGCCGGA GGGCCAAAGT ACTCCAAGCG

T  F L *
28801 TACCTTTCTG TAATCTTAGA TTAATCAGCA GAATAACCAA CCCCAAAAGC ACTTAGCACT TAGCCACACG AATCGAGTAA GTAAGTTACC AATCTCATAC

                                                                    *
28901 ACCCCCCCCA TGTACTTCTT TCTTCCATAA TTTCACTGAA TAAATAAAGA TCACAGACAA AAAAAGACAA GAGACATCGT GTGTTGTGTA ATTCGATTTA
```

Figure S3. Nucleotide sequence of the MCMV M25 ORF and deduced amino acid sequence. The nucleotide sequence of ORF M25 of the MCMV Smith strain as annotated by Rawlinson et al. (1996)[2] (Genbank accession no: NC_004065.1) is depicted. A putative TATA box and the polyadenylation signal sequence[3] are indicated as boxes. The transcription start sites and the 3´-end of the transcripts mapped in this study are labeled by arrows and a star, respectively. The putative nuclear localization signal in the M25 amino acid sequence (aa 198-204) is put between parentheses and the amino acid sequence displaying similarity to HCMV UL25 (aa 408 - 807) is underlined. The methionines that give rise to the 105 and 130 kDa M25

proteins are circled and other methionines are marked in bold. The initially proposed start codon of the M25 ORF (nt position 26,015)[2] is labeled in bold.

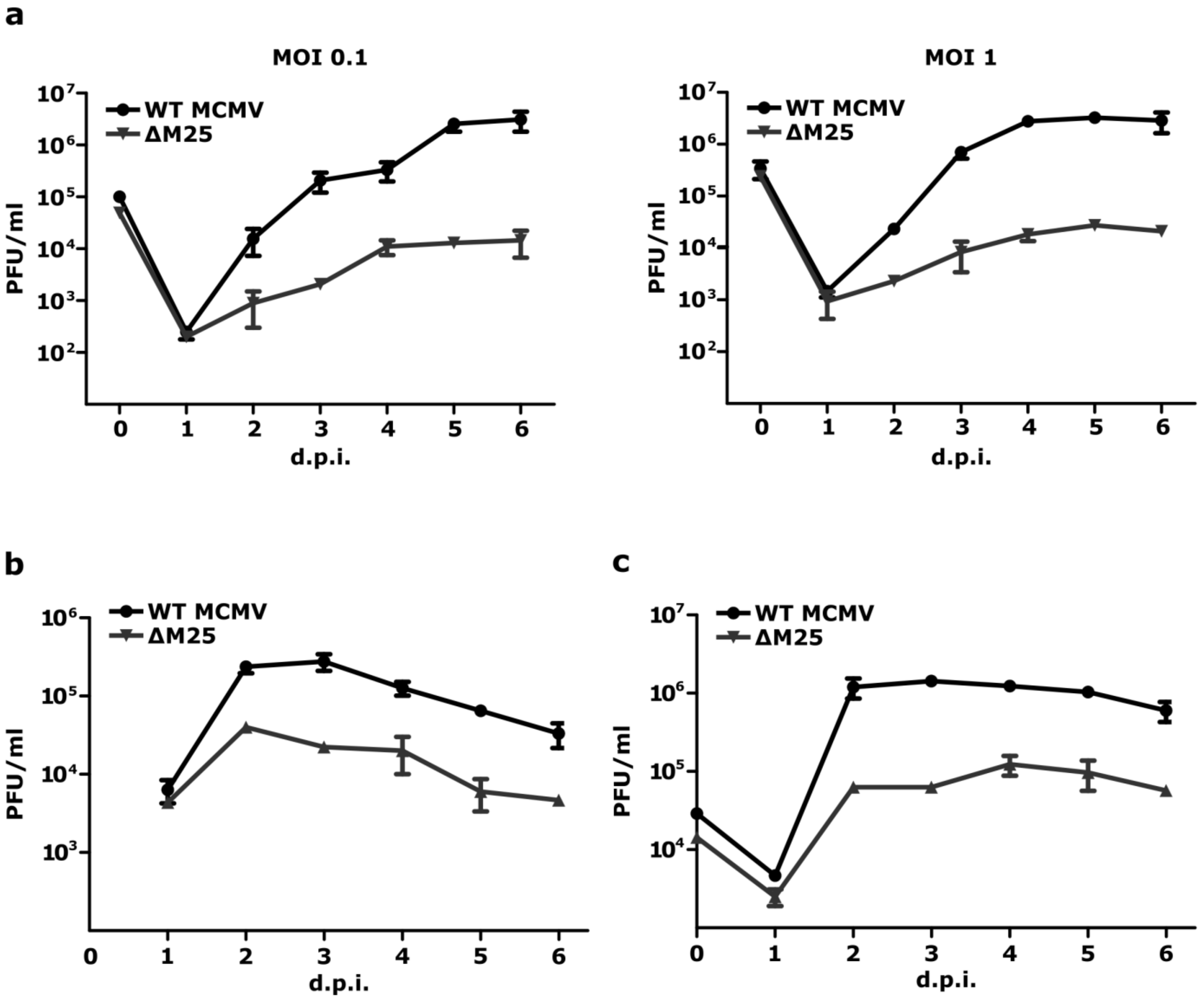

Figure S4. Growth kinetics of WT MCMV and the ΔM25 mutant. (**a**) Liver sinusoidal endothelial cells were infected with indicated viruses at MOI 0.1 or 1 and virus titers in supernatants of infected cells were determined by plaque assay. (**b, c**) MEF were infected with WT MCMV or the ΔM25 mutant at an MOI 0.5 using centrifugal enhancement. At the indicated days p.i. cells and supernatants were collected separately. Titers of cell-associated virus (**b**) and virus in the supernatant (**c**) were measured by plaque assay. Data points in graphs represent means ± SD of triplicates.

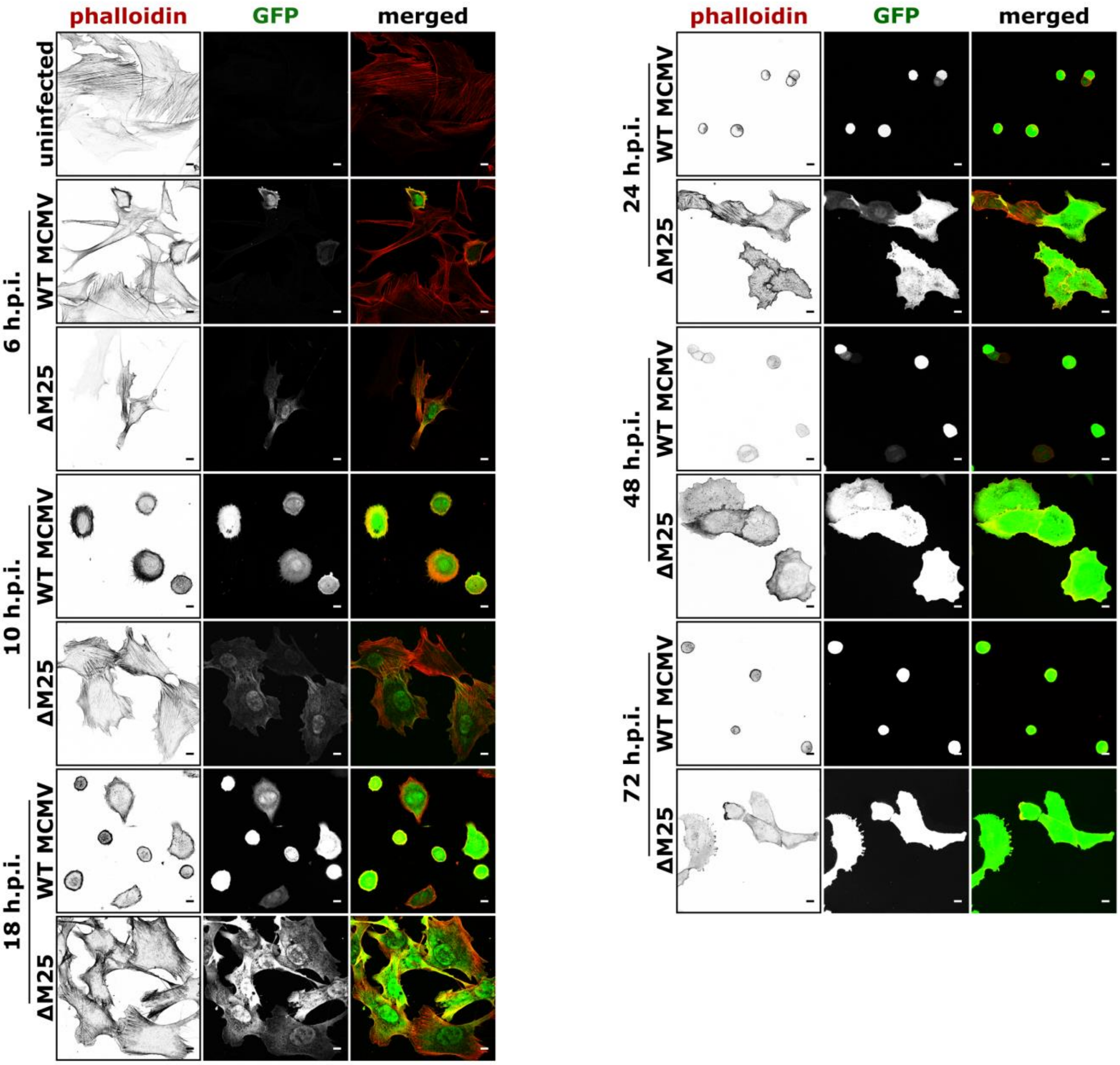

Figure S5. Overview of the morphological changes elicited by WT MCMV and the ΔM25 mutant during the course of infection. NIH3T3 were infected with indicated viruses at MOI 1 or were mock infected. At indicated time points p.i. cells were fixed, stained with phalloidin-TRITC and examined by confocal microscopy. Scale bars, 10 µm.

Supplementary Methods

Mutagenesis of MCMV BACs. Viral sequences were deleted from the MCMV BACs pSM3fr-GFP[1] and pSM3fr[4] by replacement with a PCR-amplified kanamycin resistance (knR) cassette (flanked by FRT sites) utilizing red-α, -β, -γ-mediated recombination in *E.coli* as described[5]. Where appropriate, the knR gene was subsequently excised by FLP-recombinase[5]. Primers used for construction of the different mutants are listed in supplementary table S1. Plasmid pOriM25 was generated by cloning a 4.4 kbp StuI-PstI fragment (nt 25,174 to 29,561 of the MCMV genome) into plasmid pOri6k-linker[5] and was inserted into BAC ΔM24-m25.2ΔKnR by FLP-mediated recombination[5], resulting in the BAC M25R. The MCMV BAC S-mCherry-SCP (a kind gift of J. Bosse) encoding an mCherry-tagged small capsid protein[6] served as template to delete the M25 ORF by *en passant* mutagenesis[7] using a knR cassette amplified from plasmid pOri6KanRIT (Messerle, unpublished) with primers SmCherrySCPdeltaM25f and SmCherrySCPdeltaM25r primers, resulting in BAC S-mCherry-SCPΔM25. For *in vivo* analysis the virus MCMV_GFP-P2A-ie1/3 was generated, which is based on the full-length, MCK-2-positive MCMV BAC pSM3fr-MCK-2fl[8]. To this end, an eGFP-KnR-P2A cassette was amplified with primers MCMV-iee2-prmr A#1 and MCMV_HSF1 and inserted at the 5´-end of the ie1/ie3 coding region, followed by subsequent excision of the knR marker by *en passant* mutagenesis[7]. MCMV-GFP-ie1/3 was found to replicate to comparable titers as unmodified wild-type MCMV. ORF M25 was deleted from MCMV_GFP-P2A-ie1/3 by following the same strategy as for mutagenesis of S-mCherry-SCP, resulting in BAC MCMV_GFP-P2A-ie1/3_ΔM25. The genome of the vM25GFPstop mutant was generated in analogous manner, by inserting the sequences for monomeric GFP directly downstream of the sixth ATG codon of the M25 ORF. The PCR product used for recombination was amplified with primers M25GFPstopF and M25GFPstopR using and plasmid pEP-mGFP-in (B. Sodeik, unpublished) as template.

Growth curves and plaque assay. For growth curve analysis *in vitro*, cells were infected at an MOI 0.1 or 1, and supernatants were collected daily, centrifuged for 5 min at 300 × g to remove cell debris, and frozen at – 80°C until analysis. To compare intracellular and extracellular virus yields, single-step growth analysis was performed using an MOI of 0.5, followed by centrifugal enhancement. Supernatants were harvested and cells were scraped in

medium, washed three times in PBS, re-suspended in medium and frozen at -80°C. Upon defrosting cells were disrupted by water bath sonication at 4°C (10-s pulses with amplitude of 60% until the sum of the applied energy was 4 kJ), and finally debris was removed by centrifugation for 5 min at 300 × g. Plaque assays were performed on sub-confluent MEF. Briefly, serially diluted supernatants were added to cells followed by incubation for 3 h at 37°C. Media was removed and cells were overlaid with 0.75% carboxymethyl cellulose in DMEM with 10% FCS. Plaques were detected based on virus-driven GFP expression and counted at day 5 p.i.

Analysis of post-translational modification. O-glycosylation. Mock-infected or infected NIH 3T3 cells were lysed 24 h p.i. in 50 mM Tris-HCl pH 7.5, 150 mM NaCl, 1 mM EDTA, 0.2% NP-40, 1% glycerol supplemented whit protease inhibitor cocktail (Calbiochem). Some cell samples were treated from 8 to 24 h p.i. with Thiamet G (25 µM; Santa Cruz), an inhibitor of the cellular O-GlcNAcase enzyme, to increase O-glycosylation of proteins. After lysis some of the samples were treated with β-N-Acetylhexosaminidase$_f$ (NEB) for 2 h at 37°C to remove O-linked glycans. Immunoblotting with an HA- and GlcNac-specific antibody (Cell Signaling) was performed to detect the M25 proteins and O-glycosylation, respectively.

Phosphorylation. Cells were lysed 24 h p.i. in PMP buffer (50 mM HEPES, 100 mM NaCl, 2 mM DTT, 0.01% Brij 35 pH 7.5 at 25°C; NEB) supplemented with 0.2% NP-40 and protease inhibitors cocktail (Calbiochem). Some samples were additionally supplemented with phosphatase inhibitors cocktail (Bimake). For treatment with λ phosphatase (NEB) (200 Units for 30 min at 30°C) $MnCl_2$ was added to a final concentration of 1 mM. Lysates were analyzed by immunoblotting using HA-, pERK- and GAPDH-specific antibodies. pERK was used as positive control and GAPDH as loading control.

Subcellular fractionation. Conditionally immortalized MEF were seeded in 100 mm cell culture dishes and doxycycline was removed 24 h before infection with indicated viruses. Cells were lysed at indicated times p.i. and subcellular fractions were obtained following a published protocol[9]. Briefly, cells were lysed in 500 µl of SF buffer and lysates were passed 10 times through a 24 Gauge needle. After incubation on ice for 20 min, the cytoplasmic fraction was obtained by centrifugation at 750 × g for 5 min (4°C) and further cleared by centrifugation at 10,000 × g for 10 min (4°C). Pellets were washed once in SF buffer and passed again 10 times through a 24 Gauge needle. After another centrifugation step at 750 ×

g for 5 min (4°C), the nuclear pellet was resuspended in 250 µl of NL buffer. Cytoplasmic and nuclear fractions were analyzed by immunoblotting and purity of the fractions was checked by probing membranes for lamin B or tubulin B.

Quantification of viral genomes. To determine genome copy number per cell quantification was performed by qPCR specific for the MCMV M55/gB gene and the cellular gene *pthrp* and normalization to a standard curve determined with known quantity of plasmid pDrive_gB_PTHrP_Tdy[10]. Primers and probes are listed in Supplementary Table S1. Briefly, qPCR reactions were prepared using 2 µl of a ten-time dilution of the isolated DNA, 10 µl of Brillant II qPCR Master Mix with low ROX (Agilent Technologies), 140 nmol of each primer and 50 nmol of probe. qPCR was performed using ABI 7500 RealTime PCR Machine (Applied Biosystems) controlled by ABI 7500 Software. Following initial denaturation (10 min 95°C), 40 cycles of denaturation at 95°C for 15 s, and annealing and extension at 60°C for 1 min were performed. Each sample was analyzed in triplicate and mean Ct values were used for calculating genome copy numbers.

References

1. Mathys, S. *et al*. Dendritic cells under influence of mouse cytomegalovirus have a physiologic dual role: to initiate and to restrict T cell activation. *J. Infect. Dis.* **187**, 988-999. (2003).
2. Rawlinson, W. D., Farrell, H. E. & Barrell, B. G. Analysis of the complete DNA sequence of murine cytomegalovirus. *J. Virol.* **70**, 8833-8849. (1996).
3. Dallas, P. B., Lyons, P. A., Hudson, J. B., Scalzo, A. A. & Shellam, G. R. Identification and characterization of a murine cytomegalovirus gene with homology to the UL25 open reading frame of human cytomegalovirus. *Virology* **200**, 643-650. (1994).
4. Wagner, M., Jonjic, S., Koszinowski, U. H. & Messerle, M. Systematic excision of vector sequences from the BAC-cloned herpesvirus genome during virus reconstitution. *J. Virol.* **73**, 7056-7060. (1999).
5. Borst, E. M., Benkartek, C. & Messerle, M. Use of bacterial artificial chromosomes in generating targeted mutations in human and mouse cytomegaloviruses in *Current Protocols in Immunology* (eds. Coligan, J. E. et al.,) 10.32.1-10.32.30 (John Wiley & Sons, 2007).

6. Bosse, J. B. *et al*. A beta-herpesvirus with fluorescent capsids to study transport in living cells. *PLoS One* **7**, e40585. (2012).
7. Tischer, B. K., Smith, G. A. & Osterrieder, N. En passant mutagenesis: a two step markerless red recombination system. *Methods Mol. Biol.* **634**, 421-430. (2010).
8. Jordan, S. *et al*. Virus progeny of murine cytomegalovirus bacterial artificial chromosome pSM3fr show reduced growth in salivary Glands due to a fixed mutation of MCK-2. *J. Virol.* **85**, 10346-10353. (2011).
9. Yu, Z., Huang, Z. & Lung, M. L. Subcellular fractionation of cultured human cell lines. *Bio-protocol* **3**, e754. (2013).
10. Lemmermann, N. A. W. *et al.* CD8 T-cell immunotherapy of cytomegalovirus disease in the murine model in *Methods in Microbiology* (eds. Kabelitz, D. & Kaufmann, S. H. E.) 369-420 (Academic Press, 2010).

Table S1. Oligonucleotides used in this study

A. Oligonucleotides for cloning

primer name	resulting plasmid	cloning vector	sequence 5´-3´
M25f	pM25-HA	pIRES2AcGFP1	CGGAATTCCGGCCGCCATGAGCCAGTTCGTACAGCACGTCG
M25HAr	pM25-HA	pIRES2AcGFP1	AACTGCAGAACTACGCGTAGTCCGGCACGTCGTACGGGTACAGAAAGGTACGCTT GGAGTAC
M25ATG2	pM25l-HA	pM25-HA	TGCACTGCAGCTCGAACGCCTGTCCGAGAA
M25ATG6	pM25s-HA	pM25-HA	TGCACTGCAGGAGGAACTAAACGGCGAAGA
HMIEPr	pM25l-HA<br>pM25s-HA	pM25-HA	GCGGATCTGACGGTTCACTA
BamHIM25ATG2f	pM25l	pcDNA4-myc-6xhis	GCATGGATCCGCCACCATGAACCGTCGATCCTCC
BamHIM25ATG6f	pM25s	pcDNA4-myc-6xhis	GCATGGATCCGCCACCATGTCACGCGACGGACCC
M25r	pM25l<br>pM25s	pcDNA4-myc-6xhis	GCTAGAATTCCAGAAAGGTACGCTTGGAG
M44 BamHIf	pM44	pcDNA4-myc-6xhis	GCAGGATCCGCCACCATGGAGGGTGGTAGGAAA
M44EcoRIr	pM44	pcDNA4-myc-6xhis	GCTGAATTCGGCCGCGCACTTTTGTTT
M82 HindIIIf	pM82	pcDNA4-myc-6xhis	GCAAAGCTTGCCACCATGGCCGAGGAATTTAAC
M82 EcoRIr	pM82	pcDNA4-myc-6xhis	GCTGAATTCGGGTTGTAGATGTGGGGG

B. Oligonucleotides for BAC mutagenesis

primer name	resulting virus genome	parental BAC	sequence 5´-3´
ΔM24f	ΔM24-m25.2<br>ΔM24-m25.1	pSM3fr-GFP	CCGATCTCGATGGGGCCTGCCGTGCAGTGAATCGGATAAAAATATCTGAAAGGAC GACGACGACAAGTAA
m25.2r	ΔM24-m25.2	pSM3fr-GFP	CTACGACCCGGCGCCCTACGGGGGACTATATAGGCTTGCCAACACTATGACAGGA ACACTTAACGCCTGA
Δm25.1r	ΔM24-m25.1<br>Δm25.1	pSM3fr-GFP	GTCAAGATCCGCAAGGGATGGCGGATGCCGCTGACCTGGCCCAAGAATTACAGGA ACACTTAACGCCTGA
ΔM24of	ΔM24	pSM3fr-GFP	TGGCGCCGATGCCGACGAAGCCCGGGATCGAGTCGGCTATGAGATAGTAAAGGAC GACGACGACAAGTAA
ΔM24or	ΔM24	pSM3fr-GFP	TTCGGCCGTTTCTACTGTTATCGCGGGCCCCCGGACGACGCCATCTACTACAGGAA CACTTAACGCCTGA
ΔM25of	ΔM25	pSM3fr-GFP	TCGCGACACGTCGTCGTGGACGACGACGACGACGGAGATTATGATTCACAAGGAC GACGACGACAAGTAA
ΔM25or	ΔM25	pSM3fr-GFP	GAGTAGCCTTCGAAGCCTGCCTCTTCCATGGCCGCGCACTTGATCAGATACAGGAA CACTTAACGCCTGA

Δm25.1of	Δm25.1	pSM3fr-GFP	AGGCAACGTAAGGTGCTGTTCTCCCCACAGAGATCTTGTCCTGTGAAGAAAGGAC GACGACGACAAGTAA
M25HAf	vM25HA	pSM3fr-GFP	CTCCTCCCCCGGCCGCCGGAGGGCCAAAGTACTCCAAGCGTACCTTTCTGTACCCG TACGACGTGCCGGACTACGCGTAGAGGACGACGACGACAAGTAA
M25r	vM25HA	pSM3fr-GFP	CGTGTGGCTAAGTGCTAAGTGCTTTTGGGGTTGGTTATTCTGCTGATTAACAGGAA CACTTAACGGCTGA
SmCherrySCPΔM25f	S-mCherry-SCP-ΔM25 MCMV_GFP-ie1/3_ΔM25	S-mCherry-SCP MCMV_GFP-ie1/3	TCCAAGGACCGTCGTATGTTCGTCACCGACGACTCGTCGGCAACAGCAGTCACGC AGAAGACGCATCGTGGCCGGATCTC
SmCherrySCPΔM25r	S-mCherry-SCP-ΔM25 MCMV_GFP-ie1/3_ΔM25	S-mCherry-SCP MCMV_GFP-ie1/3	ATGATGGTGGCGGGGTGCTGCTTCTGCGTGACTGCTGTTGCCGACGAGTCGTCGGT GACGTGACCACGTCGTGGAATGC
MCMV-iee2-prmr A#1	MCMV_GFP-ie1/3	pSM3fr-MCK-2fl	GATCGGCGATCATGATCATGTTGCAACTGGGTGCGGCGGGCTCCATCTCTGCCGGG CCCGGGTTCTCTTCG
MCMV_HSF1	MCMV_GFP-ie1/3	pSM3fr-MCK-2fl	GGTCTCTGTGGACATCTGTTGATGATAAAAAATTATATTTTTTAGAGAGATGGTG AGCAAGGGCGAGGA
M25GFPstopF	vM25GFPstop	vM25HA	GTCTGAGGAACTAAACGGCGAAGAGGAGGAGAGAGACGAAGGTAAGCCTATCCC TAACCCTCTCCTCGGTCTCGATTCTACGGAAGACATGGTGAGCAAGGGCGAGGAG CTG
M25GFPstopR	vM25GFPstop	vM25HA	AGTCGTCGTCCTGGCTGTGCCGGCGGGGTCCGTCGCGTGATTACTTGTACAGCTCG TCCATGCCG

C. Oligonucleotides for qPCR

gB_Taq_probe			FAM- TGCTCGGTGTA GGTCCTCTCCAAGCC - TAMRA
gB_Taq_Fw			CTAGCTGTTTTAACGCGCGG
gB_Taq_Rev			GGTAAGGCG TGGACTAGCGAT
PTHrP_Taq_Probe			FAM - TTGCGCCGCCGTT TCTTCCTC - TAMRA
PTHrP_Taq_Fw			CAAGGGCAAGTCCATCCAAG
PTHrP_Taq_Rev			GGGACACCTCCGAGGTAGCT

D. Oligonucleodties for probe construction - Northern blot

M25pf			AACGCCAACGACGACGATGA
M25pr			TGCGCGGCGGGGTCATCAGA

E. Oligonucleotides for RACE experiments

M25-1			GGATTATCGCGTATCCGTCC
M25-2			CTGTCCGCCGTACCTATTGC
M25-3			GAGCCGGTCCTGACCAAGAA
